# Imbalanced post- and extrasynaptic SHANK2A functions during development affect social behavior in SHANK2-mediated neuropsychiatric disorders

**DOI:** 10.1038/s41380-021-01140-y

**Published:** 2021-05-21

**Authors:** Ahmed Eltokhi, Miguel A. Gonzalez-Lozano, Lars-Lennart Oettl, Andrey Rozov, Claudia Pitzer, Ralph Röth, Simone Berkel, Markus Hüser, Aliona Harten, Wolfgang Kelsch, August B. Smit, Gudrun A. Rappold, Rolf Sprengel

**Affiliations:** 1 Research Group of the Max Planck Institute for Medical Research at the Institute for Anatomy and Cell Biology, Heidelberg University, Heidelberg, Germany; 2 Department of Human Molecular Genetics, Heidelberg University, Heidelberg, Germany; 3 Department of Molecular and Cellular Neurobiology, Center for Neurogenomics and Cognitive Research, Amsterdam Neuroscience, Vrije University Amsterdam, Amsterdam, the Netherlands; 4 Central Institute of Mental Health, Medical Faculty Mannheim, Heidelberg University, Mannheim, Germany; 5 Department of Physiology and Pathophysiology, Heidelberg University, Heidelberg, Germany; 6 OpenLab of Neurobiology, Kazan Federal University, Kazan, Russia; 7 Federal Center of Brain Research and Neurotechnologies, Moscow, Russia; 8 Interdisciplinary Neurobiological Core (INBC), Heidelberg University, Heidelberg, Germany; 9 Department of Psychiatry and Psychotherapy, University Medical Center Mainz, Mainz University, Mainz, Germany; 10 Interdisciplinary Center for Neurosciences (IZN), Heidelberg University, Heidelberg, Germany

**Keywords:** Neuroscience, Genetics

## Abstract

Mutations in *SHANK* genes play an undisputed role in neuropsychiatric disorders. Until now, research has focused on the postsynaptic function of SHANKs, and prominent postsynaptic alterations in glutamatergic signal transmission have been reported in *Shank* KO mouse models. Recent studies have also suggested a possible presynaptic function of SHANK proteins, but these remain poorly defined. In this study, we examined how SHANK2 can mediate electrophysiological, molecular, and behavioral effects by conditionally overexpressing either wild-type SHANK2A or the extrasynaptic SHANK2A(R462X) variant. SHANK2A overexpression affected pre- and postsynaptic targets and revealed a reversible, development-dependent autism spectrum disorder-like behavior. SHANK2A also mediated redistribution of Ca^2+^-permeable AMPA receptors between apical and basal hippocampal CA1 dendrites, leading to impaired synaptic plasticity in the basal dendrites. Moreover, SHANK2A overexpression reduced social interaction and increased the excitatory noise in the olfactory cortex during odor processing. In contrast, overexpression of the extrasynaptic SHANK2A(R462X) variant did not impair hippocampal synaptic plasticity, but still altered the expression of presynaptic/axonal signaling proteins. We also observed an attention-deficit/hyperactivity-like behavior and improved social interaction along with enhanced signal-to-noise ratio in cortical odor processing. Our results suggest that the disruption of pre- and postsynaptic SHANK2 functions caused by *SHANK2* mutations has a strong impact on social behavior. These findings indicate that pre- and postsynaptic SHANK2 actions cooperate for normal neuronal function, and that an imbalance between these functions may lead to different neuropsychiatric disorders.

## Introduction

Coding mutations in *SHANK1–3* genes have been associated with autism spectrum disorder (ASD), and *SHANK2* and *SHANK3* mutations were also identified in other neuropsychiatric disorders, including schizophrenia [1]. In addition, *SHANK2* and *SHANK3* mutations are tightly linked with intellectual disability, while *SHANK1* mutations have been identified in ASD patients with normal intelligence [2]. All three *SHANK* genes encode an evolutionarily preserved protein domain structure, which suggests that SHANK proteins have different but related functions at the excitatory synapses, especially in glutamate receptor function and assembly during development [3].

*SHANK* genes generate multiple isoforms, some of which lack several protein–protein interaction domains. The different isoforms of the SHANK proteins have been suggested to determine the different organization of the postsynaptic proteins at different developmental stages and/or different brain regions [4]. Several *Shank3* knockout (KO) mouse models targeting different isoforms have been generated, which showed diverse phenotypes on behavioral, molecular, and electrophysiological levels [4, 5]. This revealed the specific functions of different SHANK isoforms in determining how proteins are structured within the postsynaptic density (PSD) (for a review, see [6]). However, the existing gene-targeted conventional and conditional *Shank2* KO mouse models could not fully dissect the complex function of *SHANK2* and its isoforms [5]. Thus, both *Shank2*^*Δex15-16*^ and *Shank2*^*Δex16*^ KO mouse models displayed comparable ASD-like phenotypes but with different endophenotypes, and *Shank2*^*Δex24*^ mice exhibited bipolar-associated mania-like behaviors including hyperactivity and decreased repetitive behaviors (for a review, see [5]). The behavioral differences between the three mouse models were extended to the molecular, electrophysiological, and synaptic composition, as well as to the anatomical levels. Therefore, the simple suggestion of a hypomorphic SHANK does not explain the highly variable phenotypes in *Shank* KO mice and patients with *SHANK* mutations. Other putative SHANK isoforms may still be present and could play additional roles, which may have obscured the detailed functional analysis of SHANK and masked specific phenotypes in *Shank* KO mouse models [7].

Recently, several studies have suggested presynaptic functions of the SHANK protein family [8, 9], which could be overshadowed by the prominent postsynaptic changes in *Shank* KO mouse models [10]. These possible functions on the composition and operation of the axon terminals add another layer of complexity to SHANK functions and may be a component of the diverse phenotypes in patients and *Shank* KO mice. To investigate this hypothesis and dissect the pre- and postsynaptic functions, we dominated the structure and organization of the SHANK scaffold in well-defined neuronal subtypes by using the classical gain-of-function approach and overexpressed SHANK2A, a brain-specific SHANK2 isoform, or the extrasynaptic SHANK2A(R462X) variant. We generated a doxycycline (Dox)-controlled transgenic mouse line, in which the transgenic SHANK2A isoform is expressed in those brain regions that are implicated in neuropsychiatric disorders (cortex, hippocampus, and striatum) [11]. Analogously, we generated a second transgenic mouse line expressing the SHANK2A(R462X) variant found in one ASD patient [12]. In a previous in vitro study, we provided direct evidence that the C-terminal SHANK2A SAM domain is essential for the postsynaptic localization of SHANK2A: The Venus-tagged SHANK2A(R462X) that is lacking the SAM domain could only be detected in the somata, but not in the dendritic spines [13]. Since SHANK2A(R462X) expression is associated with ASD, this suggests other extrasynaptic functions of SHANK2. Already in 1998, Du et al. suggested a role of CortBP1 (now renamed SHANK2 [14]) in axonal outgrowth, growth cone mobility, and presynaptic specializations in cultured neurons [15]. Our comparative analysis of both mouse lines revealed for the first time the necessity of its protein domains to perform its effects on axonal/pre- and postsynaptic functions and on both postsynaptic and extrasynaptic dysfunction in the progression of different neuropsychiatric disorders.

## Results

### Generation of *Tg*^*SHANK2A/tTA*^ (SH-WT) and *Tg*^*SHANK2A(R462X)/tTA*^ (SH-RX) mice

**Fig. 1:**
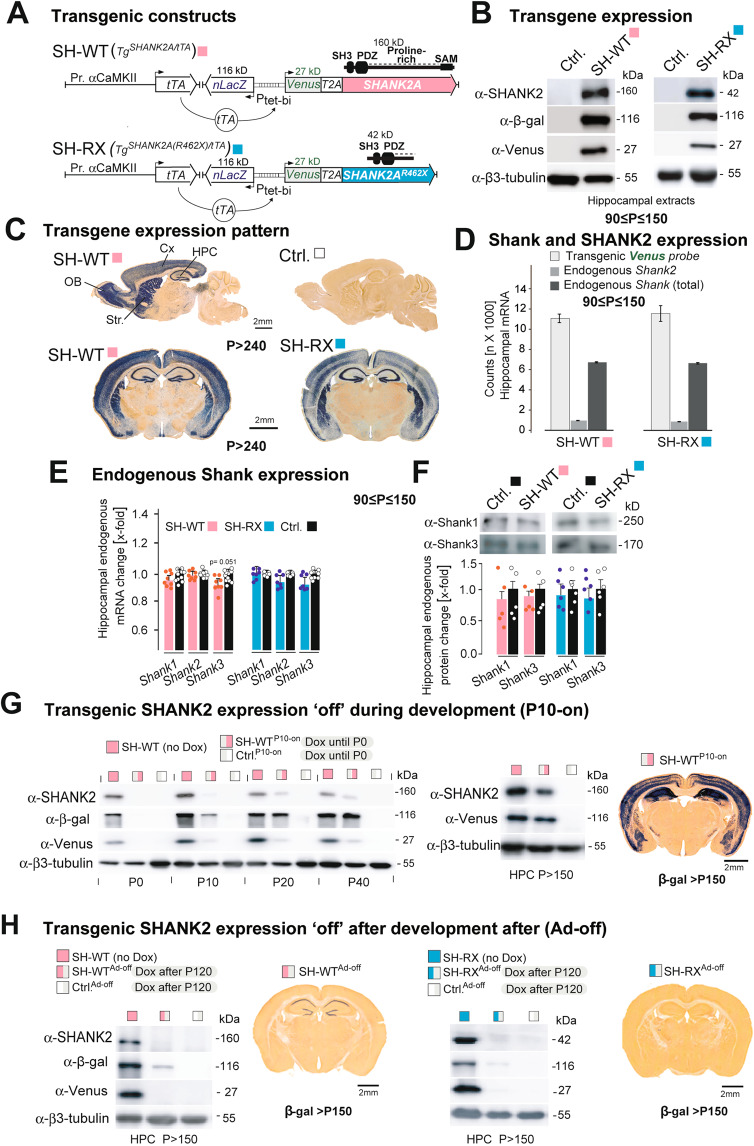
Generation and characterization of SH-WT and SH-RX mice. **A** Scheme of the two transgenes used for the overexpression of SHANK2A and SHANK2A(R462X) in glutamatergic neurons of the forebrain. The transcription activator (tTA) for the bidirectional Ptet-bi promoter of the SHANK2A and SHANK2A(R462X) transgenes can be inactivated by the presence of doxycycline (Dox). Black arrows indicate transcriptional start sites. SHANK2A isoform constitutes of 4 protein domains, whereas SHANK2A(R462X) lacks part of proline-rich and the SAM domains. **B** Hippocampal protein lysates of SH-WT and SH-RX animals showed comparable immunosignals of the transgene expressing SHANK2A and SHANK2A(R462X). The Beta-Galactosidase (β-Gal) and Venus were used as additional markers. The proteins were visualized by immunoblotting using antibodies specific to the human SHANK2, β-galactosidase, and Venus. β3-tubulin was used as a loading control. **C** top row: β-Gal activity was restricted to forebrain areas, as shown in sagittal brain sections of SH-WT mice. X-Gal on control mice showed no staining. **C** bottom row: X-Gal stained coronal brain sections from SH-WT and SH-RX mice (scale bars: 2 mm). **D** nCounter analysis revealed 12-fold mRNA expression of Venus (11,064 and 11,550 counts) compared to the endogenous *Shank2* level in the hippocampi of SH-WT (962 counts) and SH-RX (859 counts) mice, respectively. 1.6-fold higher mRNA expression of Venus compared to endogenous *Shanks* (*Shank1* + *Shank2* + *Shank3*) (6717 and 6630 counts) was measured in eight SH-WT and 7 SH-RX mice. **E** nCounter analysis revealed no change in the mRNA expression of *Shank1–3* in the hippocampi from SH-WT or SH-RX animals (*n* = 8 SH-WT and 11 control mice; *n* = 7 SH-RX and seven control mice; 3–5 months in age). **F** No change on SHANK1 and SHANK3 expression in the total protein lysates from SH-WT or SH-RX hippocampi (*n* = 5 SH-WT mice and 5 control mice; n = 6 SH-RX mice and 6 control mice; 3–5 months old). **G (left)** Dams were treated with Dox until labor (which precludes SHANK2A expression) and the brains of offspring (SH-WT^P10-on^) and littermate controls (Ctrl.^P10-on^) analyzed for residual human SHANK2A, β-gal, and Venus expression. Protein lysates of the forebrain were used at different postnatal stages (P0–P40) from SH-WT^P10-on^ and Ctrl.^P10-on^ mice. The expression of the β-gal and Venus transgenes started at around P10. **G** (**middle)** Total protein lysates of the hippocampus from adult SH-WT^P10-on^ and Ctrl.^P10-on^ mice were analyzed by immunoblotting with the human-specific SHANK2 and Venus antibodies and revealed high expression levels of SHANK2A and Venus, but lower levels compared to the positive control mice. **G** (**right)** β-gal expression of the nlacZ gene can be detected at the cellular level of adult SH-WT^P10-on^ mice by X-gal staining (scale bar: 2 mm). **H** Protein lysates of the hippocampi from adult SH-WT, SH-RX, and respective littermate control mice treated for 1 month with Dox (SH-WT^Ad-off^, SH-RX^Ad-off^, and Ctrl.^Ad-off^) were analyzed by immunoblotting using the human-specific SHANK2, β-gal, and Venus antibodies. Immunoblots revealed an almost complete absence of β-gal and complete absence of Venus and polycistronic SHANK2 expression in Dox-treated mice (7 months). Some β-gal expression of the nlacZ gene can still be detected by X-Gal staining at the cellular level in SH-WT^Ad-off^, but not SH-RX^Ad-off^ mice (scale bars: 2 mm).

For a conditional overexpression of the SHANK2A isoform in the forebrain of mice, we employed the well-established Dox-controlled Tet-Off system (Fig. 1A). Using this system, we obtained SHANK2A and SHANK2A(R462X) overexpressing mouse lines, named SH-WT and SH-RX, respectively, with comparable Dox-controlled transgene expression levels and expression patterns in excitatory neurons in the hippocampus, cortex, inhibitory medium spiny neurons (MSN) in the striatum and granule cells in the olfactory bulb (Fig. 1B, C; Fig. S1–S3). In the hippocampus, quantitative n-Counter mRNA analysis revealed a 12-fold overexpression for both bicistronic *Venus2A-SHANK2A* and *Venus2A-SHANK2A(R462X)* transcripts when compared to the endogenous *Shank2* gene-derived mRNA, and a 1.6-fold increased expression relative to the sum of all endogenous *Shanks (Shank1–3)* (Fig. 1D). Endogenous *Shank1* and *Shank3* mRNA and protein levels were neither affected by SHANK2A nor by SHANK2A(R462X) transgene overexpression (Fig. 1E, F), excluding a feedback regulation of *Shank* gene expression. To eliminate the developmental expression of the functional Camk2a-tTA (Fig. S3) and thus the developmental expression of the transgenic SHANK2As, pregnant mice received a Dox diet until labor, causing a complete switch-off of the tTA-controlled transgene expression till P10 (Fig. 1G). Dox-treatment of adult transgenic mice for at least one month triggered an efficient shut-down of the transgene expression in adult SH-WT^Ad-off^ and SH-RX^Ad-off^ mice (Fig. 1H).

### Transgenic SHANK2A expression induced a shift of Ca^2+^-permeable AMPARs in CA1 cells, and SHANK2A(R462X) increased the branching of CA1 basal dendrites

**Fig. 2:**
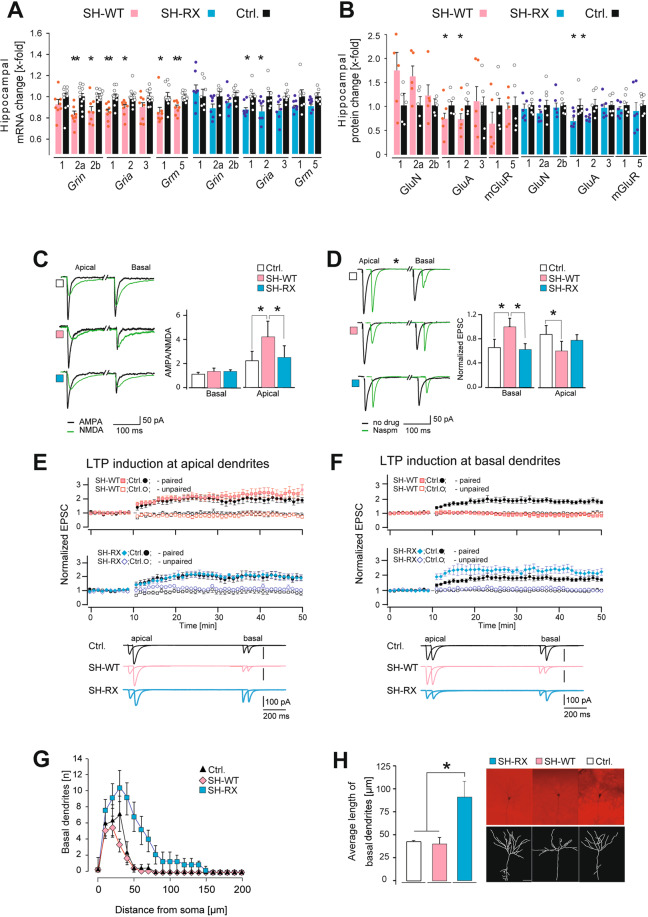
Structural alterations and AMPAR conductance imbalance in apical and basal dendrites in the CA1 hippocampal region of SH-WT and SH-RX  mice. **A** Hippocampal mRNA expression of SH-WT mice showed on average a down-regulation of NMDA (*Grin2a* and *Grin2b*), AMPA (*Gria1* and *Gria2*), and metabotropic glutamate (*Grm1* and *Grm5*) receptor genes using nCounter analysis (*n* = 8 SH-WT and 11 control mice, 3–5 months). Expression analysis of SH-RX hippocampi revealed a significant down-regulation of AMPAR genes (*Gria1* and *Gria2*) (*n* = 7 SH-RX and 7 control mice, 3–5 months). **B** Total protein lysate analysis of SH-WT hippocampi confirmed the downregulation of AMPAR subunits GluA1 and 2 (*n* = 5 mice for each genotype, 3–5 months). For SH-RX, the down-regulation of the AMPAR subunits GluA1 & 2 was also confirmed (*n* = 6 mice for each genotype, 3–5 months). Unpaired two-tailed Student’s *t* test followed by the Benjamini-Hochberg test, *p ≤ 0.05, **p ≤ 0.01. **C**. AMPA/NMDA ratio measurements revealed an increase of the ratio in SH-WT but not in SH-RX mice in the apical dendrites in the CA1 hippocampal region compared with control mice. No difference in the AMPA/NMDA ratio was found between SH-WT, SH-RX, and control mice in the basal dendrites in the CA1 hippocampal region. **D** Naspm treatment, a blocker of AMPARs lacking the GluA2 subunit, decreased the current in the apical dendrites of SH-WT mice compared to SH-RX and control mice, which showed a reduced current in the basal dendrites. **E** SH-WT and SH-RX mice revealed no difference in the pairing-induced LTP in the apical dendrites compared to control mice. **F** Synapses at basal dendrites of SH-WT mice showed no LTP and those of SH-RX mice showed a nonsignificant trend towards increased LTP compared to control mice (*n* = 6; unpaired two-tailed Student’s *t* test, *p ≤ 0.05, 4–6 weeks). **G** Sholl analysis showed an increased number of basal dendrites and **H** longer basal dendrites in CA1 pyramidal cells of SH-RX animals compared to SH-WT and control mice (*n* = 3 mice for each genotype; One-way ANOVA followed by the turkey multiple comparison test, *p ≤ 0.05). Representative pictures of biocytin-filled neurons in CA1 hippocampal regions from SH-RX, SH-WT and control mice along with the respective reconstruction of their basal and apical dendrites. Scale bar: 50 μm. Standard errors are given as SEM for **A**, **B**, **G**, **H** and as SD for **C**–**F**.

Since the postsynaptic glutamatergic system is the main focus in research on shankopathies [16] and since our study focused mainly on the well-studied synaptic transmission and plasticity of hippocampal synapses, we measured mRNA and protein expression of NMDA, AMPA, and metabotropic glutamatergic receptors in the hippocampi of SHANK2A and SHANK2A(R462X) overexpressing mice. On the mRNA level, SH-WT showed a downregulation of the NMDAR genes *Grin2a* and *2b*, AMPAR genes *Gria1* and *2*, and metabotropic glutamatergic receptor *Grm1* and *5* (Fig. 2A). Only the downregulation of *Gria1* and *2* was confirmed on the protein level (Fig. 2B and Fig. S4A). For SH-RX, the expression of *Gria1* and *2* was downregulated, which was confirmed by immunoblots (Fig. 2A, B and Fig. S4A). Whereas *Grin2a, Grin2b,* and *Gria1* expressions were normal when SHANK2A was suppressed during development or when the transgenic SHANK2A and SHANK2A(R462X) expression was suppressed in adulthood. The mRNAs for the AMPAR subunits 2,3 and mGluRs were reduced when the SHANK2A transgene was transiently expressed (Fig. S4B, C). On the other hand, suppression of SHANK2A(R462X) transgene expression in adult mice rescued the reduction of *Gria1* but not *Gria2* expression.

Despite the reduced global hippocampal *Gria1/2* mRNA and GluA1/2 protein levels in both SH-WT and SH-RX mice, electrophysiological recordings from the CA1 pyramidal cells in the hippocampus revealed regular synaptic NMDAR and AMPAR currents in basal and apical dendrites in SH-RX mice compared to controls. In the apical dendrites of SH-WT mice, a specific increase of the AMPAR response could be recorded (Fig. 2C). This selective enhancement of AMPAR-mediated EPSC was mediated by an AMPAR subtype switch from a low conductance GluA2-containing to a high conductance AMPAR subtype lacking GluA2. This was evidenced by a robust reduction of the AMPAR currents in the apical dendrites by the selective GluA2-lacking AMPAR channel blocker, 1-naphthyl acetyl spermine (Naspm) in SH-WT mice (Fig. 2D). Simultaneously, with the increased sensitivity of apical dendrites to Naspm the basal dendrites became Naspm resistant (Fig. 2D), indicating a specific subcellular reduction of Ca^2+^-permeable AMPARs in basal dendrites in SH-WT mice. The decrease of Ca^2+^-permeable AMPARs in basal dendrites could be monitored also by the lack of pairing-induced long-term potentiation (LTP), where Ca^2+^-permeable AMPARs contribute to LTP induction [17, 18]. The increased level of high conductance AMPARs in apical dendrites did not change or facilitate LTP levels (Fig. 2E, F). In SH-RX mice, as expected from the somatic expression of SHANK2A(R462X) [13], the transgenic SHANK2A(R462X) neither affected the AMPAR currents nor the AMPAR subtype in CA1 pyramidal cells (Fig. 2C, D). A trend towards a subcellular-specific increase of LTP together with an increased branching of CA1 basal dendrites in the *stratum oriens* in SH-RX mice could be monitored (Fig. 2F–H). The slightly increased CA3-to-CA1 LTP in SH-RX mice and the AMPAR subtype switch towards Ca^2+^-permeable AMPAR in apical and towards Ca^2+^-impermeable AMPAR in basal dendrites of SH-WT mice, cannot be explained by reduced GluA1 levels, indicating that the reduced glutamate receptor levels in hippocampal extract reflect the pool of extrasynaptic receptors. Thus, our electrophysiological experiments show that SHANK2A modulates the AMPAR levels on a subcellular level in synapses to modulate the synaptic plasticity, and that for this modulation the SAM domain is essential. When SHANK2 is expressed without the SAM domain, this SHANK2A loses its ability as a postsynaptic organizer scaffold protein. This provides direct functional evidence that SHANK2A(R462X) does not act as an AMPAR organizer in the postsynapse.

### Comparative proteomic and pathway analysis of the hippocampus highlights targets for axonal/presynaptic function in SH-WT and SH-RX and additional postsynaptic targets in SH-WT only

To extend the quantification of our preselected mRNA expression and immunosignal analysis (Fig. 1E, F and Fig. 2A, B), we screened more than 2000 proteins in the synaptic-enriched P2 fractions and employed a quantification by mass spectrometry (MS) combined with and ConsensusPathDB/KEGG pathway [19] and SYNGO [20] analyses to identify alterations in proteins that are involved in synaptic function. As input data, the protein-rich fraction (P2 fraction)—representing a crude purification of proteins outside of the cell soma—was used. To avoid a preselection of our protein pool to only proteins of the synaptic scone, we did not use the PSD-enriched fraction, which is strongly enriched by exclusively postsynaptic proteins.

By using the SWATH-MS acquisition approach [21, 22], a total of 2466 proteins were identified and quantified based on 9206 peptides present in all samples (Supplementary Data 1). The median coefficient of variation (Fig. S5A) of the protein quantification was 11%, 13%, and 10% in control, SH-WT, and SH-RX groups, respectively (Fig. S5B). Only minor differences in variation were observed between a protein identified with only one peptide and with two or more peptides (Fig. S5C). The statistical analysis revealed 104 and 55 proteins in adult SH-WT and SH-RX mice, respectively, with significantly different protein levels compared to controls (FDR corrected *p* < 0.05; Table S1, S2). The proteomic analysis was performed using statistics corrected for multiple testing. In SH-WT mice, the total SHANK2 level (mouse and human SHANK2A protein) was 2.4-fold increased, while Shank1 and Shank3 reached a 0.66 and 0.74-fold score of the levels compared to the synaptic-enriched Shank1 and Shank3 from control littermates (Table S1), indicating that (i) on the postsynaptic level, SHANK2A overexpression displaced Shank1 and Shank3 and that (ii) the effect of transgenic overexpression in the synaptic-enriched P2 fraction is not as strong as suggested by the total mRNA and protein levels (Fig. 1D). In SH-RX mice, the transgenic SHANK2A(R462X) expression neither changed the ratio of the endogenous, synapse-enriched Shank1, 2, or 3 nor did it affect the synaptic enrichment of other major postsynaptic proteins (Table S2), in line with the somatic and extrasynaptic localization of the SHANK2A(R462X) variant previously seen in rat primary neurons [13].

**Fig. 3:**
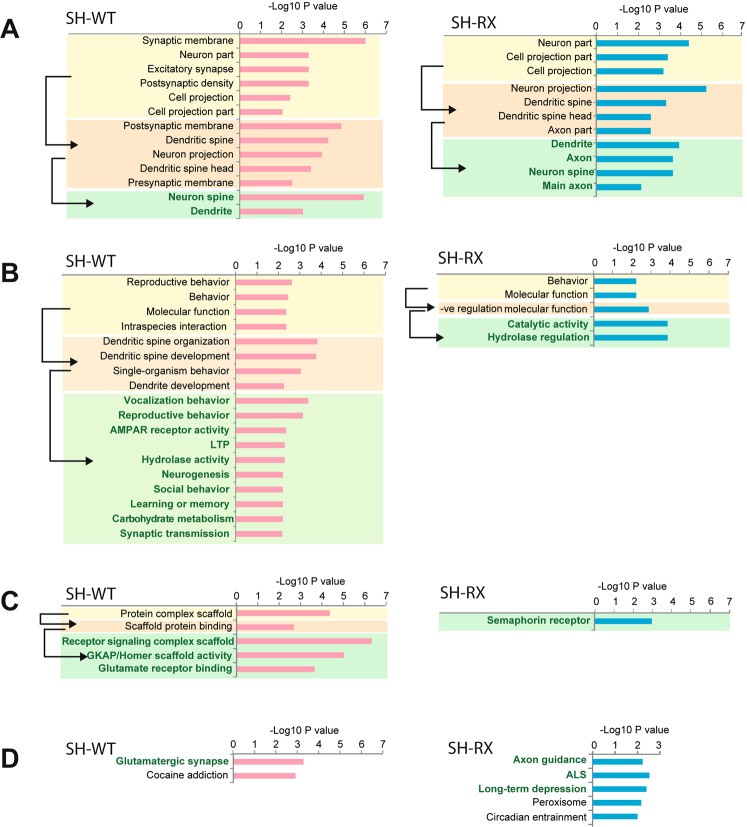
Analysis of hippocampal synapse-enriched protein fractions of SH-WT and SH-RX mice revealed line-specific alterations in pre- and postsynaptic pathways. **A** Based on the abundant proteins of hippocampal synapses, the cellular component analysis of ConsensusPathDB database proposed that the synaptic membrane, neuron part, postsynaptic density, cell projections, and neuronal spines might be affected in SH-WT and SH-RX mice. **B** The biological process analysis proposed significant alterations in the behavior, dendritic organization, and altered vocalization for SH-WT mice but not for SH-RX mice. **C** The molecular function analysis predicted alterations in the scaffold organization of glutamatergic synapses for SH-WT mice and alteration in the semaphorin receptor function for axon guidance in SH-RX mice. **D** The KEGG pathway analysis suggested that the glutamatergic synapses are affected in SH-WT, whereas the axon guidance pathway is most likely impaired in SH-RX mice. **A–C** ConsensusPathDB identified on level 2, are given in the yellow field and were eventually more specified in the third (pink) and fourth level (green). LTP = Long-term potentiation; ALS = Amyotrophic lateral sclerosis.

The cellular component and biological process analysis by “ConsensusPathDB” identified potential changes in the postsynaptic density and synaptic membrane in SH-WT and changes in axonal components in SH-RX mice. These affected pathways suggested alterations in the dendritic organization, behavior, and vocalization in SH-WT, but not in SH-RX mice (Fig. 3A, B). Similarly, the molecular function analysis, as well as the KEGG pathway analysis, predicted alterations in glutamatergic synapses in SH-WT mice. In sharp contrast, in SH-RX mice the ConsensusPathDB analysis pointed to significant alterations in axon guidance due to semaphorin receptor-controlled pathways (Fig. 3C, D).

Similarly, the enrichment analysis in SynGO [20] of protein levels identified pre- and postsynaptic changes in SH-WT and underlined the alterations in presynaptic/axonal functions and vesicular release in SH-RX mice (Fig. S6). This result was also obtained when we removed the one-hit peptides, or the SHANK proteins from the SYNGO input list of altered proteins (data not shown). Those data provide strong indications for additional SHANK2A-induced modulation of presynaptic/axonal functions most likely by promoting neuronal outgrowth and/or synapse formation. For the presynaptic action, the SHANK2A C-terminus is dispensable, whereas, for the well-described postsynaptic action, the SHANK2A full-length protein is necessary.

Concerning the presynaptic alterations, the SH-WT and SH-RX mice share 23 differentially-expressed synapse-enriched proteins in the hippocampal P2 fraction (Table S1, S2). Three of these 23 proteins, Amisyn (*Stxbp6*), R-PTP-N2 (*Ptprn2*), and Copine V (*Cpne5*), are already differentially expressed in the *Tg*^*Camk2a-tTA*^ mice compared with wild-type controls (Table S3). This can be explained by the transgenic insertion of the *Camk2a-tTA* transgene in Chr. 12, which triggered a 508 kb chromosomal deletion in Chr. 12, removing six genes including *Ptprn2* [23]. In total, the hippocampal P2 fraction of SH-WT and SH-RX mice revealed 12 and 9 altered gene products, respectively, that were already altered in the hippocampal fraction of *Tg*^*Camk2a-tTA*^ mice (Tables S1–S3). From the remaining 20 SH-WT and SH-RX gene products with different P2 levels in hippocampi, 6 proteins were detected by using only a single peptide in the MS and might need further validation [24]. Notably, from those 20 proteins, 13 proteins are involved in axonal or neurodevelopmental functions and only 7 also in postsynaptic function, whereas the contribution of differentially changed postsynaptic proteins in the P2 fraction was much more pronounced in SH-WT mice. Unexpectedly, from the major players of the glutamatergic systems, only GluA1 *(gria1)* levels were altered the synaptic-enriched fractions of SH-WT and SH-RX mice, and Homer2 *(homer2)* and mGluR3 *(Grm3)* levels were altered in the SH-WT mice.  Other major players of the glutamatergic system were detected in the pool of analyzed proteins but their protein levels were comparable to control mice.

Strikingly, in both SH-WT and SH-RX P2 protein fractions, proteins with the most significant changes compared with control fractions (Kif1a, Mycbp2, Rae1, Map7d2, and Apba2) promote axonal and neuronal outgrowth as suggested by their enhanced levels in the P2 fraction. Some of these proteins have been described as risk genes for neuropsychiatric disorders. Kif1a, for example, is a key motor protein for the efficient axonal retrograde transport of presynaptic protein components [25]. Kif1a is associated with non-syndromic intellectual disability and spastic paraplegia [26–28]. Mycbp2 belongs to the family of PHR proteins, which function in all key steps of axonal development. But dendritic arborization was also found to be affected by *Mycbp2* mutations. Thus, PHR proteins can have opposing effects on axon termination and dendrite extension in a single neuron (for a review, see [29]). A causal link between Mycbp2 variants and ASD has not been shown so far [30]. PHR proteins interact with Rae1 [31], which, like Mycbp2, is also significantly enriched in the P2 fraction of our SH-WT and SH-RX mice. Map7d2 promotes cargo transport in the axon [32]. The *Apba2* gene encodes amyloid-beta precursor protein-binding 2 (Apba2/Mint2). Apba2/Mint2 is a presynaptic adaptor protein involved in neurexin trafficking and neuronal function, and several nonsynonymous *MINT2* variants have been identified in ASD patients [33]. We did not include NDRG3 in this list, since NDRG3 is expressed mainly in the nucleus. It is known to be involved in aggressive behavior, but additional research is required to identify a potential role for NDRG3 within the nervous system [34].

Together, these data provide strong evidence for an additional, unexpected presynaptic/axonal role of SHANK2A that can promote either directly or indirectly axonal outgrowth and presynaptic function.

### SH-WT mice exhibited ASD-like  and SH-RX mice ADHD-like phenotypes

**Fig. 4:**
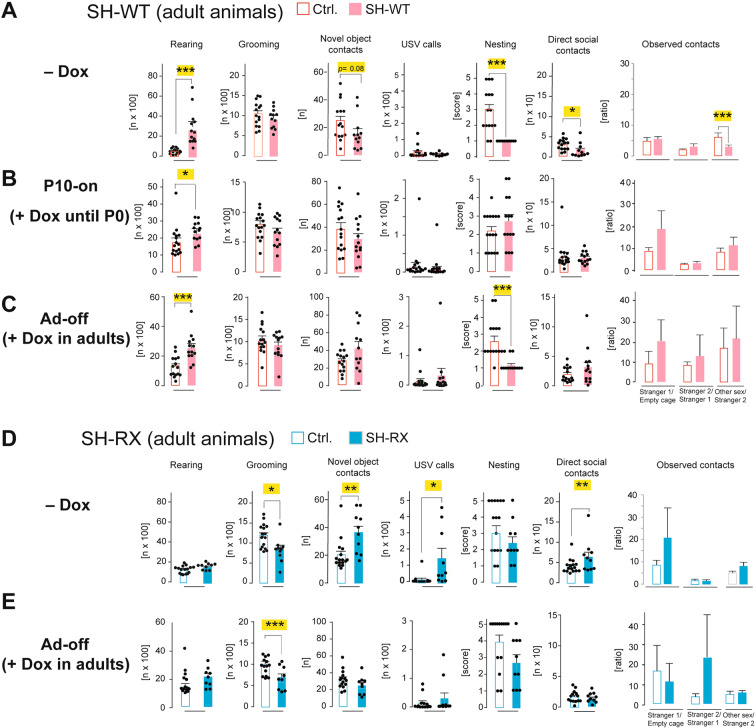
Behavioral analysis of SH-WT and SH-RX mice revealed reversible social alterations. **A** Behavioral experiments of adult SH-WT mice revealed increased repetitive rearing and normal self-grooming in the LABORAS test, a borderline significant decrease in contacts in the novel object recognition test, a normal number of USV calls, nesting impairment, reduced direct social interaction, and reduced number of contacts in the third session of the three-chamber social test (*n* = 12 SH-WT and 15 littermate control mice, 6–8 months). **B** Behavioral experiments of adult SH-WT^P10-on^ mice revealed a slight increase in repetitive rearing but no social impairment in the novel object recognition, nesting, direct social interaction, or three-chamber social tests (*n* = 14 SH-WT^P10-on^ and 16 and Ctrl.^P0-on^ mice, 4–6 months). **C** Behavioral experiments of adult SH-WT^Ad-off^ revealed increased repetitive rearing and reduced nesting behaviors but no social impairment in the novel object recognition, direct social interaction, or three-chamber social tests (*n* = 12 SH-WT^Ad-off^ and 15 Ctrl.^Ad-off^ mice, 5-8 months). **D** Behavioral analysis of adult SH-RX mice revealed normal rearing and reduced repetitive self-grooming in the LABORAS test with an enhancement of social interaction in the novel object recognition, USV, and direct social interaction tests. The nesting and three-chamber social test results were comparable to control mice (*n* = 10 SH-RX and 16 littermate control mice, 6–8 months). **E** Behavioral analysis of adult SH-RX^Ad-off^ revealed a significantly reduced number of self-grooming but normal social interaction in the novel object recognition, nesting, USV, direct social interaction, and three-chamber social tests (*n* = 9 SH-RX^Ad-off^ and 14 Ctrl.^Ad-off^ mice, 5–8 months). For all experiments, Two-way ANOVA was used (*p ≤ 0.05, **p ≤ 0.01, ***p ≤ 0.001). Error bars indicate the standard error of the mean (SEM).

To determine the effect of presynaptic dysfunction in SH-RX mice and the combined pre- and postsynaptic dysfunction in SH-WT mice on the pathophysiology of neuropsychiatric disorders, we analyzed the behavior of the different mouse cohorts. We found that SH-WT mice showed a reduced body weight and ASD-typical intensive rearing, hyperactivity, anxiety, impairment in nest building and burrowing, novel object recognition, direct social interaction, and deficits in the social preference tests (Fig. 4A and Fig. S7A, 8A). A direct causal link between transgenic SHANK2A expression and alterations in the behavioral phenotype could be demonstrated by inhibiting the transgenic *SHANK2A* transcription during prenatal and early postnatal development in SH-WT^P10-on^ mice by Dox-treatment (Fig. 1G). In adult SH-WT^P10-on^ mice, the increased rearing, hyperactivity, anxiety, burrowing impairment, and reduced body weight were strongly attenuated compared to Dox-naive SH-WT mice (Fig. 4B and Fig. S7B, 8B). Importantly, SH-WT^P10-on^ mice displayed regular nest building and novel object recognition, normalized direct social interaction, vocalization, and social preference in the three-chamber social test (Fig. 4B). Thus, the transgenic SHANK2A overexpression during development is causally linked to the social impairments in ASD.

By turning off transgenic SHANK2A expression in adult animals (SH-WT^Ad-off^; Fig. 1H), we found that SH-WT^Ad-off^ mice still displayed hyperactivity, increased anxiety, and alterations in rearing, burrowing, and nest building (Fig. 4C and Fig. S7C, 8C). However, the social impairment and novelty interaction of SH-WT^Ad-off^ were comparable to controls (Fig. 4C), which illustrates that regarding social behavior, the neurodevelopmental SHANK2A dysfunction in the neuronal network can be reversed, as shown for both SHANK2 [35] and SHANK3 [36].

In SH-RX mice, the hyperactivity, anxiety, and burrowing impairment were less pronounced compared to SH-WT mice (Fig. S7D, 8D). But in sharp contrast to SH-WT mice, the repetitive self-grooming was reduced in the night and the light phases and novel object recognition, vocalization, and direct social interaction were significantly enhanced compared to littermate controls (Fig. 4D). Again—analogous to SH-WT mice—the switch-off of the SHANK2A(R462X) overexpression in adult mice (SH-RX^Ad-off^) triggered a rescue of the enhanced social activities (in this case attenuation of hypersociablity) but failed to normalize the hyperactivity, burrowing impairment or reduced self-grooming (Fig. 4E and Fig. S7E, 8E).

The cognitive function of the mice was tested in the hippocampus-dependent Puzzle box task [37] and in the classical fear conditioning task. The SH-WT mice showed mild impairment in the memory-dependent executive function and no impairment in fear memory, whereas the SH-RX mice showed poor memory in both tasks (Fig. S9).

To assess if the hyperactivity in SH-RX mice resembled that of ADHD or bipolar-associated mania, we treated both SH-WT and SH-RX mice  with amphetamine, which attenuates the hyperactivity associated with ADHD while exacerbating the mania of bipolar disorder [38]. By amphetamine treatment, we could attenuate the hyperactivity of SH-WT and SH-RX mice significantly, whereas it induced the expected transient mania-like hyperactivity in controls (Fig. S10A,B). Accordingly, we conclude that the hyperactivity in SH-RX mice is consistent with an ADHD-like phenotype. Although the same neuronal circuits that increase hyperlocomotion may be affected by transgenic SHANK2A and SHANK2A(R462X) expression, the neuronal ensembles directing social behavior showed a reverse response towards novelty and social activities, when either SHANK2A or SHANK2A(R462X) were transgenically expressed. Since Dox-treated control littermates without the SHANK2A or SHANK2A(R462X) transgene expression served as respective controls, behavioral effects by the potential anti-inflammatory activity or liver toxicity of Dox [39] can be excluded. In addition, in one of our previous studies,  control mice with prenatal Dox treatment showed regular brain anatomy and solved several complex memory tasks [40].

### Information processing in the olfactory cortex is distorted bi-directionally in SH-WT and SH-RX mice

The opposite social behavior of SH-RX and SH-WT mice suggested that neuronal responses to social novelty are differently affected in SH-RX and SH-WT mice. Rodents use olfaction for the primary sensing of social recognition and interaction by evaluating the incoming stream of multimodal sensory information. In this information stream, the anterior olfactory nucleus (AON)—as part of primary olfactory cortices of the limbic system—controls state-dependent coding during social interactions and is critical to both recognition and exploration of conspecifics [41–43]. Changes in sensory encoding in mouse models related to neurodevelopmental and neuropsychiatric disorders have been reported for higher-level regions such as the prefrontal cortex (e.g. [44]). It is not clear whether related changes occur already in the primary sensory cortices in mouse models for ASD.

**Fig. 5:**
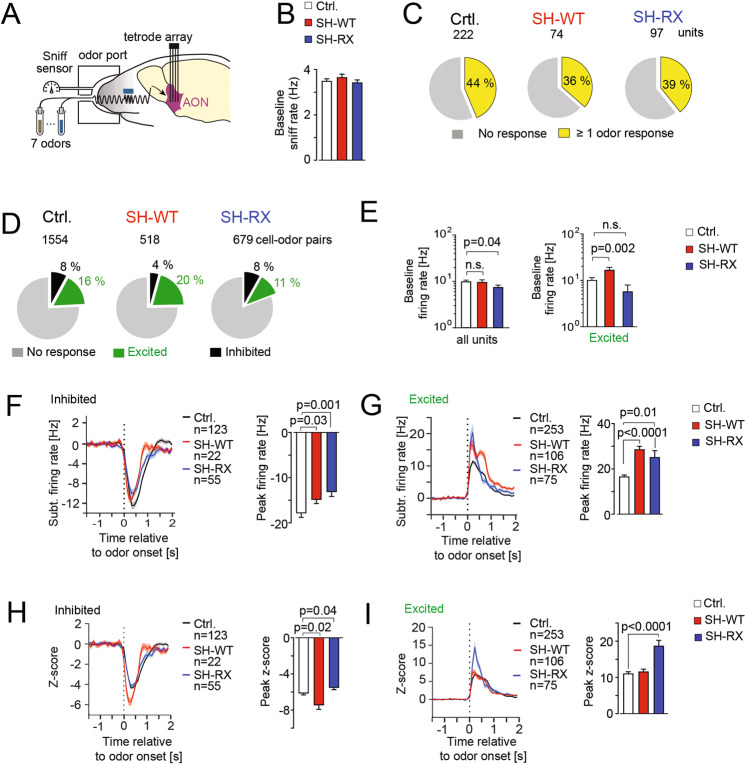
SH-WT and SH-RX mice display altered E/I ratios and signal-to-noise in cortical stimulus encoding. **A** Mice were implanted with an array for chronic recordings being placed bilaterally in the anterior olfactory nucleus (AON). Seven aldehyde odorants with increasing carbon chain length Propanal to Nonanal were applied for 500 ms in pseudo-randomized trials during recordings with 16 tetrodes in awake head-fixed mice. **B** The baseline sniff frequency was comparable between the three genotypes (ANOVA: *p* = 0.45; 31 sessions in 6 Ctrl. mice, 11 sessions in 4 SH-WT, and 24 sessions in 6 SH-RX mice). **C** Percentage of units that were recruited by at least one of the 7 odorants with a significant response (−1.96 > z-score > 1.96) tended to be decreased for SH-WT and SH-RX mice (for unit features, see Fig. S11A). **D** The distribution of significant excitatory or inhibitory responses (−1.96 > z-score > 1.96) in all cell-odor pairs from units shown in **C** differed among the genotypes. **E** Left, mean baseline firing of all units was decreased in SH-RX mice (data were log-transformed for statistical tests, ANOVA, Dunnett’s post-test, *p* < 0.05; Ctrl.: 222, SH-WT: 74, SH-RX: 97). Right, mean baseline firing of excited units was increased in SH-WT mice (data were log-transformed for statistical tests, ANOVA, Dunnett’s post-test, *p* = 0.0002; Ctrl.: 58, SH-WT: 21, SH-RX: 18). Data are shown on a logarithmic scale. Inhibited units are not shown due to the low number of units in SH-WT mice that were inhibited (*n* = 6). While **F** peak inhibitory odor responses were decreased in SH-WT (ANOVA: *p* < 0.0001; Ctrl.: 123, SH-WT: 22, SH-RX: 55 cell-odor pairs), **G** the baseline subtracted mean excitatory peak responses increased both in SH-WT and SH-RX mice (ANOVA: *p* < 0.0001; Ctrl.: 253, SH-WT: 106, SH-RX: 75 cell-odor pairs) (Dunnett’s post-test as indicated, respectively) (bin size 100 ms). The signal-to-noise was expressed as the z-score of the odor responses to baseline in all cell-odor pairs. **H** The z-score of inhibitory responses increased or decreased depending on the genotype (ANOVA: *p* = 0.0007; Ctrl.: 123, SH-WT: 22, SH-RX: 55 cell-odor pairs) (Dunnett’s post-test as indicated, respectively). **I** Note that for excitatory responses, the mean z-score only increased in SH-RX mice (ANOVA: *p* < 0.0001; Ctrl.: 253, SH-WT: 106, SH-RX: 75 cell-odor pairs), due to the involvement of the background activity in the z-score calculation. For all panels, significances are indicated as ns: *p* > 0.05, *p ≤ 0.05, **p ≤ 0.01, and ***p ≤ 0.001.

We, therefore, aimed to investigate the opposite social phenotype in SH-WT and SH-RX mice by examining the processing of pseudo-randomized presentation of seven molecularly similar odorant cues in single-unit recordings from the AON in awake, head-fixed mice (Fig. 5A). Specifically, we examined if the excitation/inhibition (E/I) balance in the network representations of olfactory stimuli was altered. We examined the sensory coding in the whole population consisting of both inhibited and excited AON neurons (Fig. S11A). The baseline sniffing frequency and the fraction of neurons with a response to at least one of the odorants were comparable between all genotypes (Fig. 5B, C). Also, the broadness of tuning, i.e., the number of odorants that evoked either an odor-excited or inhibited response in cell-odor pairs was comparable (Fig. S11B). However, the fraction of E/I odor responses differed among genotypes (Fig. 5D**;** Fig. S11B). Across all cell-odor pairs, the E/I ratio of odor responses was 2.0 in controls, 5.0 in SH-WT, and 1.4 in SH-RX mice (Fig. 5D). Thus, the stimulus excited activity of AON neurons was increased in SH-WT mice and decreased in SH-RX mice. The baseline firing (2 s before odor onset) across all units was decreased in SH-RX mice (Fig. 5E). AON neurons with odor-excited responses displayed a significantly increased baseline activity in SH-WT mice (Fig. 5E), indicating that the E/I ratio in the background noise is increased in SH-WT, while in SH-RX mice the network drive is globally reduced.

Importantly, also the AON unit responses to odors were differentially affected (Fig. S11C, D). The mean population odor-inhibited responses were smaller in the two transgenic genotypes compared to controls (Fig. 5F and Fig. S11E), and the peak-firing increases of task-excited responses were larger in SH-WT and SH-RX mice (Fig. 5G and Fig. S11E). While subtle bidirectional changes in the signal-to-noise ratio of stimulus-inhibited responses occurred in the two transgenic lines (Fig. 5H), the signal-to-noise for odor-excited responses in SH-WT remained unchanged (Fig. 5I) as the increased excitatory peak firing coincided with higher background noise compared to control mice. Yet, the signal-to-noise ratio of excitatory responses increased in SH-RX mice (Fig. 5I), due to their simultaneous increase in peak firing and decrease in background activity. In addition, in SH-RX mice, the peak width was narrower than in controls (Fig. S11F). Together these findings support a more precise sensory encoding in stimulus-excited olfactory cortex units of SH-RX mice, while SH-WT displayed an increased E/I balance of sensory coding in a system with more noise.

## Discussion

Here we show that the brain-specific SHANK2A isoform has a direct effect on (I) daily life activities, anxiety, and social-related behaviors, (II) the molecular composition and plasticity of hippocampal synapses, (III) extrasynaptic functions, and (IV) the neuronal network in the anterior olfactory nucleus during development. The side-by-side comparative analysis of exogenous SHANK2A and the extrasynaptic SHANK2A(R462X) in excitatory forebrain neurons dissected the post- and extrasynaptic actions of SHANK2A at the molecular, physiological and behavioral level (Table 1A). In “Decipher V10.0” database (https://decipher.sanger.ac.uk), several patients with ASD, intellectual disability and ADHD were reported with a duplication/triplication spanning the SHANK2 gene and in experimental systems, a *Shank3* transgenic mouse model, modeling a human SHANK3 duplication, exhibited a mania-like phenotype and seizures consistent with synaptic excitatory/inhibitory imbalance [45]. Those data show that the disturbance of the balanced SHANK expression leads to neuropsychiatric phenotypes, emphasizing that the genetically controlled, endogenous SHANK expression is essential for the establishment and/or expression of higher brain functions such as social and cognitive behaviors.

**Table 1:** **A**. Effects of pre-and postsynaptic localized SHANK2A and SHANK2A(R462X) overexpression in transgenic mice. **B**. Schematic representations of the distribution of synapses enriched for Ca^2+^-permeable (in green; high conductance) or Ca^2+^-impermeable (in orange; low conductance) AMPARs in apical and basal dendrites of hippocampal CA1 pyramidal cells in wild-type, SHANK2A overexpressing (SH-WT) and SHANK2A(R462X) overexpressing (SH-RX) mice.

In our experimental approach, we achieve a 2.4-fold overexpression of the exogenous human SHANK2A in hippocampal neurons compared to the endogenous Shanks. Despite this overexpression, the hippocampal GluA1 levels and electrically-stimulated AMPAR current responses in CA1 pyramidal cells were not reduced. Instead, the SHANK2A overload in hippocampal excitatory neurons (i) increased the synaptic AMPAR response at apical CA1 dendrites by the incorporation of high-conductance Ca^2+^-permeable AMPAR and (ii) decreased their synaptic presence at basal dendrites (Table 1B). Thus, the developmental expression of the endogenous SHANK2 had a significant impact on the fundamental mechanism underlying synaptic maturation and the establishment of neural circuits during development. This SHANK2-mediated reorganization of selective AMPAR types in synapses finds support by results in hippocampal cultures. During synaptic maturation and plasticity, a Zn^2+^-dependent switch from GluA2-lacking (Ca^2+^-permeable) to GluA2-containing (Ca^2+^-impermeable) AMPAR has been documented when Shank2 and Shank3 cooperate [3]. Moreover, the involvement of GluA1 in neuropsychiatric disorders is well described [46, 47].

The marginally increased LTP at basal CA1 dendrites by exogenous SHANK2A(R462X) might be due to a GluA1-independent LTP component that is operative in young mice when GluA1 levels are still low [48]. Since SHANK2A(R462X) is not localized in the PSD [13], neither the Shank1–3 levels nor the postsynaptic signaling proteins in the synaptic-enriched fraction (with few exceptions such as GluA1 and Nos1) are altered. However, and unexpectedly, the analysis of the hippocampal synapse-enriched proteomic fraction in presence of exogenous SHANK2A(R462X) in excitatory neurons identified several proteins involved in transmitter release and axonal outgrowth which might explain the increase of basal dendrites. Among those synaptic enriched proteins, 23 proteins  were also differentially expressed in the hippocampal synaptic-enriched proteome of thetransgenic SHANK2A, supporting an underestimated axonal function of SHANK2A and adding a functional explanation to the presynaptic/axonal localization of SHANK proteins [8, 9]. Our data suggest a direct presynaptic/axonal SHANK2 activity during early development, in addition to the post-to-presynapse SHANK2 communication, as postulated for the SHANK2-modulated neuroligin/neurexin signaling [49]. Among the presynaptic differentially-regulated proteins, *Plxna1* and *Plxna4* were less abundant. PLXNA1 and PLXNA4 are known to signal the binding of semaphorins in a short-distance inhibitory manner. The Semaphorin/Plexin signaling has multiple functions in the regulation of both axonal and dendritic differentiation and branching [50–52] and may account for the increased branching of basal dendrites of CA1 neurons in presence of additional SHANK2A(R462X). Cortical neurons from induced pluripotent stem cells derived from ASD-affected donors with SHANK2 mutations showed an increase in dendritic length and complexity as well as synapse number [53]. Similarly, SHANK3 is known to participate in growth cone motility in developing neurons. Mutations in *SHANK3* strongly affect the development and morphology of dendritic spines, reduce synaptic transmission in mature neurons, and also inhibit the effect of SHANK3 on growth cone motility [54].

It is well established that the SAM domains of SHANK proteins can bind to each other in a homomeric and heteromeric manner, enabling the SHANK proteins to multimerize tail-to-tail by a Zn^2+^-dependent mechanism [55, 56]. The SAM domain is also essential for the localization of SHANK2 and SHANK3 proteins to the PSD [57]. As SHANK2A(R462X) lacks the SAM domain, SHANK2A(R462X) does not contribute to the postsynaptic scaffold and structural organization of postsynaptic receptors. Although we identified several axonal and presynaptic and putative downstream targets of SHANK2A(R462X), its direct presynaptic action remains speculative. The presence of the SH3 and PDZ domains might be sufficient to participate in the functional molecular organization of presynaptic or active zone proteins that contain PDZ and/or SH3 protein binding sites. The SHANK binding to one of the three SH3 domains of RIMBP2, might promote RIM to tether Ca^2+^ channels to the presynaptic active zone and thus to regulate Ca^2+^ secretion coupling [58] or it might participate in the priming of synaptic vesicles via the recruitment of Munc13-1 [59].

Similarly, the trafficking and function of AMPARs are controlled by auxiliary proteins and adaptor proteins. For instance, the interaction between the Rho-GAP protein Rich2 and SHANK3 can regulate AMPAR recycling and trafficking [60]. SHANK proteins could induce such modulations of the trafficking of GluA1-type (Ca^2+^-permeable) AMPARs via the adaptor protein Rich or by the increasing synaptic accumulation of GluA2-type (Ca^2+^-impermeable) AMPARs via the glutamate receptor-interacting protein [3]. Our unexpected finding that SHANK can regulate this trafficking on a subcellular level adds another layer of complexity to the field of activity-dependent AMPAR modulation that includes receptor trafficking and AMPAR auxiliary subunits [61, 62].

We cannot exclude that some of the observed axonal/presynaptic SHANK2A-mediated alterations were induced by retrograde signaling. However, since the transgenic extrasynaptic SHANK2A(R462X) mutant has an identical effect on axonal proteins, this is very unlikely; a bifunctional role of (i) the PSD-localized SHANK2A and (ii) the cytoplasmic SHANK2A seems to be most likely. Whether these dual pre- and postsynaptic functions are specific to SHANK2A and only important during development of the nervous system and maturation of axons and dendrites remains to be resolved. It is likely that some of the molecular effects we observed are not induced directly by SHANK2A but are secondary consequences of impaired or inhibited neuronal maturation induced by the transgenic SHANK2A variants in SH-WT and SH-RX mice.

Although the SHANK2A(R462X)-associated ADHD-like hyperactivity, anxiety, and impaired daily life activities, and the increased social and reduced repetitive behaviors were diametrically opposed to the SHANK2A-induced poor social interaction and enhanced repetitive behavior [5], all social behavioral impairments of adult SH-WT mice could be attributed to the unbalanced SHANK2A expression during the development in excitatory neurons. The expression in the inhibitory granular cells of the olfactory bulb did not alter the odor recognition. However, the hyperactivity and the opposed response of SH-WT and SH-RX mice to amphetamine, when compared to control animals, was most likely attributed to the transgenic SHANK2A or SHANK2A(R462X) expression in MSN in the striatum, even if alterations of striatal circuits might also have an effect on typical ASD-like behaviors [63, 64].

Balancing the SHANK2A expression during late postnatal development and in adults attenuated the hyperactivity, anxiety, and food burrowing and normalized the social impairment. This finding underlines the strong developmental component in genetically inherited ASD. It also suggests that after postnatal development, SHANK1 and SHANK3 dominate the SHANK scaffold in the mature nervous system, as recently evidenced by the very pronounced neurophysiological phenotype of *Shank1/3* double KO mice and the early death of 60% of these mice within the first five weeks of life [65]. It also excludes a major direct contribution of transgenic chromosome 12 insertions [23] of the *Camk2a-tTA* transgene in our behavioral analysis. By normalizing the SHANK2 levels in adults, the sociability could be normalized. Similar to the genetic rescue of *Shank3* KO mice [36], only social and novelty interactions were restored, demonstrating that the neuronal circuits for social responses are not permanent and that the SHANK scaffold in neuronal circuits for neuronal circuits can be remodeled in adults genetically or by pharmacological modulation of the synaptic transmission [35].

The very different impact of the postsynaptic and isolated presynaptic SHANK2A activity on neuronal network activity of SH-WT and SH-RX mice was directly correlated with differences in odor encoding in the olfactory cortex. The exogenous SHANK2A promoted the formation of stimulus-excited units in the AON as recognized by the increased excitability, in the absence and presence of an odor stimulus. In contrast, SHANK2A(R462X) reduced the basal excitability signaling, improved the signal-to-noise ratio of odor cues with direct consequences for the social activity. Thus, the more precise sensory encoding in stimulus-excited olfactory cortex units of SH-RX mice, and the increased E/I balance of sensory coding in a system with more noise is in line with the opposing social behavior in SH-RX and in SH-WT mice. It supports the recent finding of the different c-Fos expression and the opposing social behavior of a *Shank2* and a *Shank3* KO mouse line. This analysis demonstrated that the social behavior is critically determined by the balanced excitation/inhibition ratios in a subset of brain regions that collectively contribute to the abnormalities in social dominance and social cooperation that is differently affected in *Shank2* and *Shank3* KO mice [66].

In summary, our study has revealed that SHANK2A modulates the proteomic composition and plasticity of excitatory synapses during pre- and postnatal development when endogenous SHANK2 expression reaches its peak [67]. In the presynapse, the SHANK2A-regulated proteins are well-known synaptic and axonal  organizers. In the postsynapse, Ca^2+^-permeable AMPARs were reorganized at synapses of basal and apical dendrites, which modulated their plasticity. In excitatory olfactory cortex units, SHANK2A-modulated signaling was a critical component of social behavior. Increased excitatory noise in the AON units by exogenous SHANK2A was associated with decreased social interaction. Improving the precision of excitatory cortical coding in the AON units by selective presynaptic SHANK2A alterations, in turn, came with increased social interests. SHANK2A exerted its effect on the social nervous system during pre- and early postnatal development only. Therefore, we conclude that the SHANK2A postsynaptic modifications are accompanied by less pronounced but cooperative presynaptic modifications that can fine-tune the synaptic efficiency and neuronal connectivity.

## Materials and methods

### Ethical statement

Mice maintenance and procedures were performed according to the animal welfare guidelines of the Max Planck Society and the Heidelberg University. Transgenic animals were generated by pronucleus injection into fertilized C57BL6/N oocytes (license number AZ: 35-9185.81/G-219/11; Governmental Council Karlsruhe). In vivo recordings were performed under the license (AZ: 35-9181.81/G-239/14) to W. Kelsch. All behavioral experiments were performed at the Interdisciplinary Neurobehavioral Core Facility of Heidelberg University (INBC) under the license (AZ: 35-9185.81/G-100/16) to C. Pitzer. SH-WT mice are bred and housed under the license (AZ: 35-9181.81/G-328/19) of the Regierungspräsidium Karlsruhe due to their reduced body weight and increased mortality several weeks after birth. Mice killed for postmortem analysis were registered at the IBF/Heidelberg University (T-61/15, T-51/17 and T48/19).

### Animals

For the generation of the conditional expression of SHANK2A in mice,  we used the well-established doxycycline-controlled expression system [68–70]. The tTA responder transgenic mice were generated by the pronuclear injection of C57BL/6 blastocytes with either SHANK2A or SHANK2A(R462X) plasmid fragments that encode the bi-directional tetracycline-responsive promoter element (Ptet-bi) [71] controlling the expression of the nuclear-localized β-galactosidase (nLacZ) on one side and fusion transcript composed of green fluorescent protein variant (Venus), fused *via* the ribosomal skipping sequence T2A to either SHANK2A or SHANK2A(R462X) on the other side (Fig. 1A). To activate the expression of the tTA-dependent genes and direct it towards the forebrain neurons, the responder transgenic mice were crossed with *Tg*^*Camk2a-tTA*^ mice (activator mice) in a C57BL/6N background that expresses the dox-sensitive transactivator (tTA) under the control of an 8.5 kb fragment of the αCaMKII promoter [72], which led to the generation and selection of *Tg*^*SHANK2A/tTA*^ and *Tg*^*SHANK2A(R462X)/tTA*^ double transgenic mice with similar nLacZ expression levels (Fig. 1A, C). These lines, expressing tTA in addition to either SHANK2A or SHANK2(R462X), are named *Tg*^*SHANK2A*^ (SH-WT) and *Tg*^*SHANK2A(R462X*)^ (SH-RX), respectively. Littermate mice with a single transgene, either the tTA-activator or the responder transgene, were included as control littermates together with non-transgenic littermate control mice (Ctrl.). For adult SH-WT as well as SH-RX mice, the expression of either SHANK2A or SHANK2A(R462X) along with other reporter proteins is strictly dependent on the synthetic transcription factor, the “tetracycline transactivator” (tTA). In adult Dox naïve mice, the expression of transgenes can be found in the excitatory neurons of the hippocampus and cortex [73], inhibitory granular cells in the olfactory bulb [68], and inhibitory MSN in the dorsal striatum [74] (Fig. 1C; Fig. S1–S3). In our previous experiment using the Camk2a–tTA expression system, we noticed already in mouse embryos and at P0 a substantial expression of functionally active Camk2a-controlled tTA transgenes [68, 73] (Fig. S3), which is also detectable by the β-galactosidase, Venus, and transgenic SHANK2 expression in brain extracts of SH-WT mice (Fig. 1G). After the Dox clearance in the newborn pups, the Camk2a-tTA-controlled β-galactosidase, Venus and SHANK2A reached a substantial expression level 3 weeks after birth (Fig. 1G). In contrast to Tetracycline, which normally leads to cleft palate and congenital abnormalities, there is no evidence that Dox is teratogenic [75]. Nevertheless, it is well documented that Dox has anti-inflammatory effects (e.g., [76, 77]). Therefore, in all Dox experiments, littermates treated with Dox were used as control mice to discard any effect possibly induced by the Dox treatment. Mice were housed in the IBF at Heidelberg University under a 12 h light-dark cycle and given access to water and food ad libitum. One week before the behavioral analysis, the mice were transferred to t~he INBC without changing the light-dark cycle for the animals. Dox treatment was performed at the INBC. To stop the transgene expression in *Tg*^*SHANK2A/tTA*^ and *Tg*^*SHANK2A(R462X/tTA)*^ adult mice, Dox (Sigma-Aldrich) at a concentration of 2 g/l, supplemented with 5% sucrose, was dissolved in water and provided to adult mice in light-protected bottles. To avoid tTA-controlled transgene expression during neurodevelopment in offspring, 50 mg/l dox, supplemented with 5% sucrose, was provided in water to pregnant mice until labor.

### Eosin/X-Gal staining

Enzymatic activity of β-galactosidase expression was assessed by Eosin/X-Gal staining following the standard protocol with modifications. Brain slices were washed in PBS for 20 min and then incubated in LacZ solution (0.5 M K_4_Fe(CN))_6_, 0.5 M K_3_Fe(CN)_6_, 0.2 M MgCl_2_, X-Gal 20 mg/ml in PBS) for 2 h at 37 °C. The slices were then washed three times with 1× PBS and shortly washed in 10 mM TRIS (pH 7.6) before being mounted onto glass slides. After an overnight dry session in RT, 0.5% Eosin solution was used as a counterstain, and slides were shortly washed in demineralized water, 70% EtOH, 80% EtOH, and 100 % EtOH, respectively. After drying, slides were washed in Xylol, and drops of Eukitt were put onto each slice. As the last step, the coverslips were placed onto the slices and the slides were left to air dry at RT for polymerization.

### Immunoblot analysis

All steps for total protein lysate preparation from different brain tissues were performed at 4 °C. Cortex, hippocampus, olfactory bulb, and cerebellum of the mouse brain were collected and homogenized in ice-cold buffer (25 mM HEPES, pH 7.4) containing a protease inhibitor cocktail (Complete, Roche, Pharma AG). After 5 min of centrifugation at 2000 rpm, the supernatant containing the total amount of proteins was collected. Protein concentration was determined with the BCA protein assay kit (Pierce). Ten mg of protein were separated by SDS–PAGE (8–12% separating and 4% stacking gels) and transferred to Amersham^TM^ nitrocellulose membranes Protran(R) (Sigma-Aldrich). Membranes were probed with the antibodies which are given in Table S4 together with the dilution used. Chemiluminescence was developed using a detection reagent (GE Healthcare, Amersham^TM^, ECLTM Prime Western Blotting Detection Regent, RPN2232) and visualized with the Fujifilm LAS-3000 Luminescent Image Analyzer (Object No.: B00000623) using the Image Reader LAS-3000 software and quantified using ImageJ software. As exemplified for the anti-human SHANK2, the anti-beta-tubulin antibody and anti-Venus antibody (Fig. S2C), the quality of the antibodies was tested before usage.

### Messenger RNA Expression nCounter analysis

Total RNA from mice hippocampi was extracted with TRIzol (Invitrogen) and the gene expression profile was investigated at the nCounter Core Facility, Heidelberg, using the nCounter Dx analysis system GEN1 (NanoString Technologies). A customized elements code set with 12 target genes and 6 reference genes was applied (for probe design, see Table S5). The detailed workflow is described at https://www.nanostring.com/support/product-support/supportworkflow. Background correction and normalization of data were performed using the nSolver Analysis Software 3.0 (NanoString Technologies). Positive control and reference gene normalization were performed according to the gene expression analysis guideline from NanoString Technologies (https://www.nanostring.com/application/files/7715/1251/5220/Gene_Expression_Data_Analysis_Guidelines.pdf; accessed June 2018). All the chosen reference genes *Gapdh*, *Sdha*, *Hprt1 Gpi1, Hspd1*, and *Pgk1* showed a stable expression and were selected for normalization based on the geNorm method [78]. The unit of measurement is “codeset counts”. The codeset counts of the control mice were set to 1. Original data are accessible at https://edmond.mpdl.mpg.de under (Collections: Sprengel).

### Electrophysiological analysis

Transverse hippocampal 300 μm slices were prepared from the brains of mice at the age of 4–7 weeks. Mice were killed by cervical dislocation. The slicing chamber contained an oxygenated ice-cold solution composed of (in mM): *K*-Gluconate, 140; *N*-(2-hydroxyethyl) piperazine-*N*′-ethanesulfonic acid (HEPES), 10; Na-Gluconate, 15; ethylene glycol-bis (2-aminoethyl)-*N, N, N*′*, N*′-tetraacetic acid, 0.2; and NaCl, 4 (pH 7.2). Slices were incubated for 30 min at 35 °C before being stored at room temperature in artificial CSF (ACSF) containing (in mM): NaCl, 125; NaHCO_3_, 25; KCl, 2.5; NaH_2_PO_4_, 1.25; MgCl_2_, 1; CaCl_2_, 2; and D-glucose, 25; bubbled with 95% O_2_ and 5% CO_2_. During experiments, slices were continuously perfused with the same ACSF. Patch electrodes were pulled from hard borosilicate capillary glass (Sutter Instruments flaming/brown micropipette puller). Electrodes for the postsynaptic pyramidal cells were filled with a solution consisting of (in mM): Cs-gluconate, 100; CsCl, 40; HEPES, 10; NaCl, 8; MgATP, 4; MgGTP, 0.3; phosphocreatine, 10 (pH 7.3 with CsOH).

CA1 pyramidal cells were visually identified using IR-video microscopy. Whole-cell recordings from these neurons were taken at room temperature (23–25 °C) in voltage-clamp mode using a HEKA EPC-7 amplifier (List Elektronik) with a sampling rate of 100 μs and filtered at 3 kHz. EPSCs were evoked from two independent inputs, basal and apical dendrites, with two patch pipettes as stimulating electrodes located in *str. oriens* and *str. radiatum*, respectively. The two stimulus pipettes were >200 μm apart, located below and above the soma of a CA1 pyramidal cell. All measurements were at –70 mV membrane potential.

The AMPA/NMDA current ratios were measured in Mg^2+^-free ACSF. AMPA- and NMDA-mediated EPSCs were pharmacologically isolated by sequential bath application of D-APV (200 μM 2-amino-5-phosphonovaleric acid; Sigma-Aldrich) and NBQX (10 μM; Sigma Aldrich), respectively. First, the compound AMPAR and NMDAR-mediated current were recorded in Mg^2+^-free ASCF. After collecting at least 100 sweeps, the AMPA-mediated component was blocked by the application of NBQX. Then, additional 100 sweeps of the putative NMDA-mediated currents were collected and the NMDA nature was confirmed by a subsequent application of D-APV. The AMPA-mediated component was then obtained by subtracting the averaged NMDA-mediated currents from the averaged compound responses. For subsequent analysis, the mean amplitude of the AMPA currents was normalized to the level of the amplitude of the NMDAR EPSCs. When specified, 200 μM 1-naphthyl acetyl spermine (Naspm) (Tocris) was used to block Ca^2+^-permeable AMPARs [79]. In LTP experiments the control pathway was measured by stimulating synapses of the basal dendrites when apical dendrite input was potentiated and vice versa: the input in the *str. radiatum* was used as a control pathway when the paired pathway was input to synapses of the basal dendrites. LTP was evoked and recorded by voltage-clamping the membrane potential of the postsynaptic pyramidal cell to 0 mV for 3 min while stimulating the paired pathway every 1.5 s [80]. The measured amplitudes were normalized to the mean EPSCs before pairing. The NMDAR dependence was tested in the presence of 100 μM D‐APV.

### Biocytin labeling and Sholl analysis

After electrophysiological analysis, the slices having biocytin-labeled neurons (internal patch solution contained 0.1% w/v biocytin (Biomol)) after electrophysiological analysis were treated with 1% H_2_O_2_ for 10 min to quench endogenous oxidase activity. The slices were then permeabilized using 0.2% Triton in PBS for 2 h and incubated in Vectastain ABC complex for another 2 h. The slices were then washed twice with Tris 20 mM PH 7.6 for 10 min and incubated in DAB solution for 20 min in the dark. At last, 0.01% H_2_O_2_ solution was added to the well-containing slices in DAB solution and the reaction stopped by transferring the slices to a well with Tris 20 mM PH 7.6. Z-stack images of 0.1 μM intervals were taken using the Leica DMI4000 B microscope and Leica application suite advanced fluorescence software. Sholl analysis was completed in Fiji (ImageJ 2.0.0-rc-69/1.52i). Basal dendrites were traced and morphology was analyzed using the Simple Neurite Tracer (v.3.1.3 plugin) to obtain measurements for total dendrite length and Sholl analysis (3.7.4 2018-09-25) with 10 µm radius. For the representation of pictures in Fig. 3H, dendrites reconstruction was performed using Imaris ×64 9.0.2 software (RRID:SCR_007370).

### Mass spectrometry (MS) proteomic analysis

The pre- and postsynaptic protein-containing synapse-enriched fractions (P2 fractions) were prepared as previously described [81]. In brief, hippocampi from 7 SH-WT, 7 SH-RX, and 14 control mice (both non-transgenic and mice with a single transgene) were dissected and stored at –80 °C. Each tissue sample was homogenized (in 5 mM HEPES, pH 7.4, 0.32 M sucrose, protease inhibitor cocktail (Roche)) and centrifuged at 1000 × *g* for 10 min at 4 °C. The supernatant was centrifuged at 20,000 × *g* for 30 min at 4 °C, and the resulting pellets (P2 fractions) were processed for MS analysis.

In-solution digestion of proteins was performed by using the filter-aided sample preparation protocol [82]. Briefly, 30 μg of each sample were mixed with 75 μl 2% SDS, 1 mM Tris (2-carboxyethyl)phosphine and incubated at 55 °C for 1 h. Cysteines were blocked with 0.5 μl 200 mM methyl methanethiosulfonate and 200 μl 8 M Urea in Tris pH 8.8 were added. Samples were transferred to Microcon-30 filter tubes (Millipore) and centrifuged at 14,000 × *g* for 15 min at RT. Samples were washed four times with 200 μL 8 M urea and, subsequently, four times with 200 μl 50 mM ammonium bicarbonate. Proteins were digested with 0.7 μg Trypsin/Lys-C Mix (MS grade, Promega) in 50 mM ammonium bicarbonate overnight at 37 °C. Peptides were recovered by an additional wash with 200 μl 50 mM ammonium bicarbonate, dried in a SpeedVac, and stored at −20 °C until used.

For SWATH-MS, peptides were subjected to micro LC MS/MS using an Ultimate 3000 LC system (Dionex, Thermo Scientific). Peptides were trapped in a 5 mm Pepmap 100 C18 column (300 μm i.d., 5 μm particle size, Dionex) and fractionated in a 200 mm Alltima C18 column (100 μm i.d., 3 μm particle size). The concentration of acetonitrile in 0.1 % formic acid was increased linearly from 5 to 18% in 88 min, to 25% at 98 min, 40% at 108 min, and to 90% in 2 min, at a flow rate of 5 μl/min. Peptides were electro-sprayed into the TripleTOF 5600 mass spectrometer (Sciex), with a micro-spray needle voltage of 5500 V. Each SWATH cycle consisted of a parent ion scan of 150 msec and 8 Da SWATH windows (80 msec scan time), throughout a 450–770 m/z mass range, as described by [83]. The orignial data of the analysis are available in Supplementary Data 1.

The data were analyzed using Spectronaut (Version 12.0.20491.4.23092) with a spectral library previously generated from synaptosomal preparations by data-dependent acquisition [84]. Cross-run normalization was enabled and the peptide abundances were exported for further processing using R language. Limma R package was used to Loess normalize protein abundance (“normalizeCyclicLoess” function) [85, 86]. Only high confidence peptides were used for protein quantification by setting a threshold of *Q*-value of ≤ 10^−3^ (allowing one outlier within each group), while the standard was 10^−2^. We opted for this more stringent peptide selection to increase the reliability in the quantification of all proteins, while at the same time, including proteins covered by one peptide (Fig. S5). Protein abundances were obtained by the summation of the normalized peak area of their respective peptides. Identifying differentially expressed proteins is a task in proteomic studies commonly carried out simply using t-tests. In this manuscript, we demonstrated how better results can be achieved by using moderated t-statistics from the empirical Bayes procedure Limma. Statistical analysis was performed at the protein level.

### Pathway analysis

The pathway analysis was performed at the internet portals Synaptic Gene Ontologies, SynGo (https://www.syngoportal.org) [20] and ConsensusPathDB-mouse, CPDB (http://cpdb.molgen.mpg.de/MCPDB) using the integrated databases [19].

### Behavioral assays

One week prior to the start of the behavioral experiments, the mice were single-housed in an animal room with constant temperature (22 °C) and ad libitum access to water and food. They were handled 5 days extensively (10 min per mouse a day) to reduce anxiety and to get acquainted with the testing environment. Behavioral studies were performed during the light phase between 9 a.m. and 6 p.m., except for nesting and burrowing tests. The experiments were performed on 4–8 months old adult male and female mice. Littermate mice with a single transgene (either activator or responder transgene) were included in the control population along with non-transgenic control mice. All behavioral tasks were performed in a blind manner.

The behavioral studies were performed in the following order: LABORAS, open field, dark-light box, burrowing, nesting, three-chamber social, novel object recognition, neophobia, and direct social interaction. Before experiments and between different trials, all equipment was cleaned and wiped with 70 % ethanol and allowed to evaporate completely. Automatic video-taped tracking tests (Sygnis Tracker, Sygnis) were used.

### LABORAS

The LABORAS test (Laboratory Animal Behavior Observation Registration and Analysis System) is an advanced and non-invasive system that automatically recognizes several different behaviors of mice by analysis of the forces that are induced by their movement (METRIS). Each mouse was tested individually in a cage on the system for 24 h to detect the duration and frequency of locomotion, immobility, climbing, rearing, self-grooming, drinking, and eating.

### The nesting test

The mouse was placed in a new home cage at 5 p.m. with cotton nesting material. After the nest was built it was checked the next day at 7 a.m., and its quality was assessed with a complexity score from 1 (no nest) to 5 (complex nest with a wall surrounding the mouse) [87].

### The three-chamber social test

The test was performed as described previously [88], with some modifications. A social interaction box (Harvard Apparatus) divided into three compartments was used. The social arena included a transparent box (42 × 60 cm) with two transparent sliding doors that divided the left, right, and center chambers (42 × 20 cm). In the first 5 min session, the tested mouse was placed in the central chamber with open sliding doors to offer access to the other two chambers for habituation. In the second session, an empty cylindrical cage and another cylindrical cage housing an unfamiliar C57BL/6N mouse of the same sex and age as the tested mouse were located in the corners of the left and right chambers. The tested mouse was placed in the central chamber and allowed to explore the arena for 5 min. Once this session was completed, another unfamiliar C57BL/6 N mouse (novel mouse) with the same sex and age as the tested mouse was put in the empty cylindrical cage. The tested mouse was then allowed to explore the arena for 5 min. In the fourth session, the mouse in the cylindrical cage from session 2 was replaced by an unfamiliar C57BL/6N mouse of the opposite sex. The tested mouse was then allowed to explore the arena for 5 min. The location of the cages was alternated between tests. The number of observed contacts of the tested mice was counted manually.

### Novel object recognition

Each mouse was placed in the corner of a new arena (40 × 40 × 40 cm) and allowed to explore the arena for 5 min. The mouse was then returned to its home cage for 1 min and reintroduced to the arena for another 5 min with a fixed novel object (cube) in the center. The number of contacts with the object was counted manually.

### Direct social interaction test

The test mouse was placed in a white acrylic open-field box (40 × 40 × 40 cm). After 1 min, a same-sex, similar-age, unfamiliar C57BL/6 N mouse was added to the arena. The number of contacts between the tested and the unfamiliar mouse was counted manually for 5 min.

### Ultrasonic vocalizations (USV)

USV was recorded during the direct social interaction tests. USV recording (from 0 to 100 kHz) and analysis were conducted with the equipment and software from Avisoft Bioacoustics (Berlin) as described [89]. Acoustic signals were recorded, amplified, and digitized at 250 kHz with a 16-bit resolution by the Ultrasound Gate 416 Hb USB audio device, and ultrasonic condenser microphones CM16/CMPQ were placed 30 cm above the testing area.

### In vivo recordings

Recordings were performed from the pars centralis of the anterior olfactory nucleus (AON) in mlib toolbox6 control, 4 SH-WT, and 6 SH-RX mice. Each custom-built recording array contained up to 16 tetrodes. Tetrode tips were gold-plated to achieve a lower recording impedance (target: 300 kOhm at 1 kHz) with a NanoZ-device (Multichannelsystems). To connect to the Intan RHD2164 head stage during recordings, a custom-built adaptor from the Molex SlimStack connector to two 36 Omnetics Nano Strip connectors was used to connect to an Intan RHD2164 head stage amplifier. For the implantation of the recording array, mice were anesthetized with isoflurane, and pre- and post-surgery analgesia was administered. A circular skin patch above the skull was removed. Local anesthesia was applied to the skull. The lateral and nuchal muscle insertions were left intact. The operative field was then prepared by attaching the margins of the remaining skin to the circumference of the top of the skull with VetBond (3 M), thus protecting soft tissue from damage, contamination, or necrosis and leaving only the surface of the skull exposed. Holes were drilled in the skull above the regions where tetrodes were inserted and for grounding above the cerebellum. The skull was then coated with Super-Bond C&B (Sun Medical), following the insertion of a small Neuralynx gold pin as ground connected with the recording array through an insulated copper wire. The tetrode array was slowly lowered into the brain with a motorized micromanipulator. The center of the tetrode bundle was targeted from Bregma to anterior 3.0 mm and lateral 0.8 mm, reaching ventral 3.3 mm from the dorsal brain surface. After reaching the target depth, dental cement (Kulzer Palladur) was applied at the margins of the recording array so that gravity and capillary force ensured complete filling of the narrow gap between the bottom of the array and the adhesive-coated skull. Animals recovered in their home-cage. The entire surgical procedure took approximately 1 h. Animals were normally fit and eating after less than 30 min. 3–6 weeks after recovery, the mice were placed in the head-fixation setup. The first three sessions were brief (5–20 min) and served to habituate the animals to head fixation. For odorant delivery, a custom-built air-dilution olfactometer was used [90]. Odorants were kept in the liquid phase (diluted 1:100 in mineral oil) in dark vials and mixed into the nitrogen stream that was further diluted 1:10 into a constant air stream in the olfactometer. The following aldehyde odorants of increasing c-chain lengths were used in the experiments: Propanal (CHO3), Butanal (CHO4), n-Pentanal, (CHO5), Hexanal (CHO6), Heptanal (CHO7), Octanal (CHO8), Nonanal (CHO9) (Sigma-Aldrich CAS n. 123-38-6, 123-72-8, 110-62-3, 66-25-1, 111-71-7, 124-13-0, 124-19-6, respectively). Odorants were each delivered 30 times in a pseudo-randomized order with a maximum of 3 consecutive presentations of the same odorant. Odorants were applied for 500 ms with an inter-trial interval of 10 s. Recordings were performed with an Intan 64 channel RHD 2164 miniature amplifier board connected to a RHD2000 interface board and open-source Intan interface software. Inputs from the olfactometer and sniff sensor were simultaneously recorded with the same interface board. Data were sampled at 30 kHz. To monitor the sniff signal, we used a custom-built snout mask that was gently pressed against the snout to generate a cavity in the mask in which pressure fluctuations were continuously measured through an HDI pressure sensor HDIM020GBY8H3 (First Sensor Inc.) connected to the analog input of the RHD2000 interface board (Intan). The mask design is modified based on the original design of Dmitry Rinberg (personal communications). The influx of odorized air into the cavity of the mask was calibrated to the outflow through a continuously measured vacuum. The animals quickly adapted and tolerated the pressure mask. After completion of the experiments, animals were euthanized and perfused with 4% PFA. Heads were severed with the implants attached and subsequently soaked in 4% PFA for up to 4 weeks. Due to tissue shrinkage, the exact location of the tetrode tip could not be determined, but revealed preserved electrode tracts in sagittal 150 µm sections.

### Data processing of in vivo recordings

For the pre-processing of the recorded data and the spike detection, we subtracted the median voltage trace of all channels from each recorded trace. The resulting signal was bandpass filtered between 300 and 5,000 Hz (4th order Butterworth filter, built-in MATLAB function). A threshold value for spikes was computed as a multiple (7.5×) of the median absolute deviation of the filtered signal [91]. Temporally proximal detected peaks over threshold were pruned by height to a minimum distance of 1 ms to avoid multiple detections of the same multiphasic spike. When an event was detected on multiple channels of a tetrode, the timestamp of the highest detected peak was used. Spike waveforms were extracted around −10 to +21 samples around the peak. Spike sorting was done with a custom-built graphical user interface in MATLAB, originally developed by A. Koulakov (CSHL). Metrics used for clustering included detected peak height or amplitude (and the respective principal components over channels) and the first three principal components of the waveforms for each respective channel when a spike was predominantly recorded on one channel. The quality of single unit clusters was assessed using the mlib toolbox by Maik Stüttgen (Vs. 6, https://de.mathworks.com/matlabcentral/fileexchange/37339-mlib-toolbox-for-analyzing-spike-data) with particular attention to peak height distribution (fraction of lost spikes due to detection threshold), contamination (fraction of spikes during the refractory period <5 ms) and waveform variance. The experimenter performing the recordings, spike sorting, and clustering was blinded to the genotypes of the mice.

For further analyzes, units were only included if they complied with a set of criteria: Throughout the analyzed part of the recording session, units were allowed to only have a maximum change in baseline firing rate from beginning to the end of the session of less than 10% and intermittent maximum fluctuations of 20%. Only units with a baseline firing rate of at least 0.5 Hz were included for further analysis. Clustering units into putative principal and fast-spiking neurons based on their spike waveform and baseline firing rate do not match the observed features of AON single units (Fig. S11A). Therefore, we examined the whole population of AON neurons together.

Z-scored firing rates were obtained by subtracting the mean baseline firing rate (window of 2 s before odor onset) from the mean firing rate of each bin and dividing by the baseline standard deviation computed across all trials. For baseline subtracted firing rates, the mean firing rate in a window of 1 s before odor onset was calculated for each cell-odor pair and subtracted from the whole PSTH. For group statistics, Welch’s ANOVA test and Dunnett’s multiple comparison post-tests were indicated (GraphPad Prism8). The post-test was computed in relation to control mice.

### Analysis

For nCounter- and protein quantification (Fig. 2A, B), the unpaired two-tailed student’s t-test was used, and the P values were corrected for multiple tests using the Benjamini–Hochberg test. For the cellular physiology (Fig. 2C–F), the paired two-tailed student’s *t* test was used to compare the responses from the paired vs. unpaired input. Data between genotypes were compared by the unpaired two-tailed student’s *t* test and given as means ± SD. For the Sholl analysis (Fig. 2G, H), One-way ANOVA followed by the turkey multiple comparison test was used. Data were analyzed using Prism6 software (GraphPad, https://www.graphpad.com). All data are shown as mean ± SEM. For the proteomic analysis, empirical Bayes moderated t-statistics with multiple testing correction by false discovery rate was used (“eBayes” and “topTable” functions from Limma R package). For the behavioral analysis (Fig. 4), the two-way ANOVA test was used to compare the transgenic and control mice using sex and genotype as interacting factors. For the in vivo physiology (Fig. 5), firing rate data of responses were normalized by either subtracting the baseline from the response or by *z*-scoring the PSTH. The data are shown as mean ± SEM. The sample sizes of each statistical analysis were: Fig. 5B: 31 sessions in 6 Ctrl. mice, 11 sessions in 4 SH-WT, and 24 sessions in 6 SH-RX. Fig. 5E: all units: Ctrl.: 222, SH-WT: 74, SH-RX: 97; and all excited units: Ctrl.: 58, SH-WT: 21, SH-RX: 18. Fig.  5F: Ctrl.: 123, SH-WT: 22, SH-RX: 55 cell-odor pairs. Fig. 5G: Ctrl.: 253, SH-WT: 106, SH-RX: 75 cell-odor pairs. Fig. 5H: Ctrl.: 123, SH-WT: 22, SH-RX: 55 cell-odor pairs. Fig. 5I: Ctrl.: 253, SH-WT: 106, SH-RX: 75 cell-odor pairs. The following statistical tests were used for each analysis for the in vivo physiology: Fig. 5B: ANOVA, Dunnett’s post-test, *p* = 0.45, Fig. 5E: left: ANOVA, Dunnett’s post-test, *p* < 0.05, right: ANOVA, Dunnett’s post-test, *p* = 0.0002, Fig. 5F: ANOVA, Dunnett’s post-test, *p* < 0.0001, Fig. 5G: ANOVA, Dunnett’s post-test, *p* < 0.0001, Fig. 5H: ANOVA, Dunnett’s post-test, *p* = 0.0007, Fig. 5I: ANOVA, Dunnett’s post-test, *p* < 0.0001.

## Supplementary information

Supplementary Information

Supplementary data 1 (Dataset 1)

## Data Availability

Proteomics data are available in the Supplementary Data 1 (Dataset 1) https://edmond.mpdl.mpg.de.
