# Supplementary material for: Imbalanced post- and extrasynaptic SHANK2A functions during development affect social behavior in SHANK2-mediated neuropsychiatric disorders
Source: Mol Psychiatry. 2021 May 21;26(11):6482–504. doi: 10.1038/s41380-021-01140-y (PMC8760046; doi:10.1038/s41380-021-01140-y)
Supplement: Supplementary file 1 — Supplementary Information [file 41380_2021_1140_MOESM1_ESM.pdf]

Supplementary Information

Imbalanced post- and extrasynaptic SHANK2A functions during development affect social behavior in SHANK2-mediated neuropsychiatric disorders

Ahmed Eltokhi et al., 2021

Supplemental Figures
Figures S1 – S11

Supplemental Tables
S1) Proteomics data for (SH-WT, SH-RX and Ctrls.)
S2) Proteomics data for (SH-RX and Ctrls.)
S3) Proteomics data (*Tg*$^{Camk2a\text{-}tTA}$ vs. wild-type Ctrls.)
S4) List of used antibodies
S5) nCounter probes

Proteomics data are available in the Supplementary Data 1 (Dataset 1)

Supplemental Methods
Supplemental References

SH-RX (P>90)

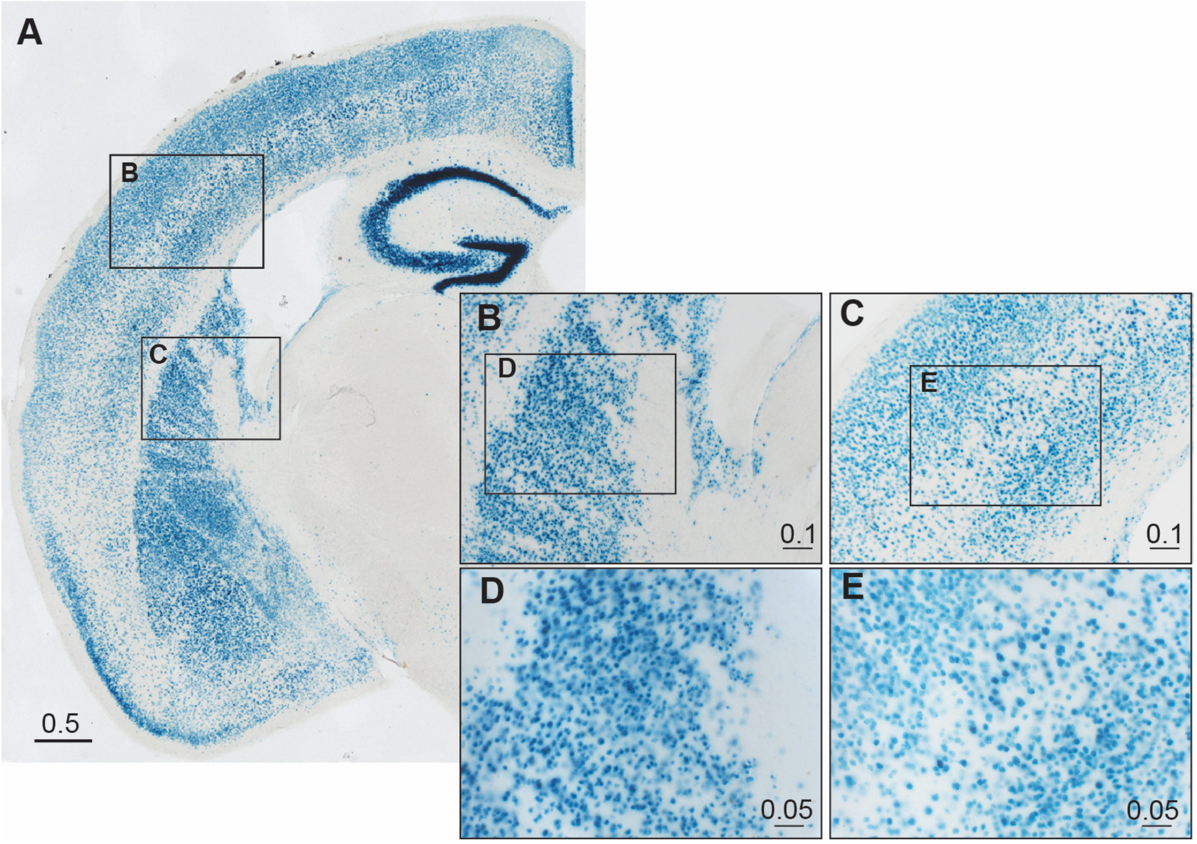

Fig. S1. Expression pattern of the SHANK2A(R462X) transgene as monitored by the co-regulated nuclear-localized β-galactosidase. The nuclear β-galactosidase activity monitored by X-Gal staining is shown in a coronal brain section of an adult SH-RX mouse (C57Bl6/N; $Tg^{(PtetO\text{-}nlacZ\text{-}SHANK2A(R462X)}/Tg^{Camk2a\text{-}tTA}$). The intensity of the X-Gal identifies the CA1 pyramidal, DG granular cells and cells in the piriform cortex as neuronal cell types with the strongest expression of $Tg^{Camk2a\text{-}tTA}$ driven PtetO-nlacZ-SHANK2A$^{(R462X)}$. Lower expression was found in other forebrain regions including the amygdala and striatum. Scale bars are given in mm.

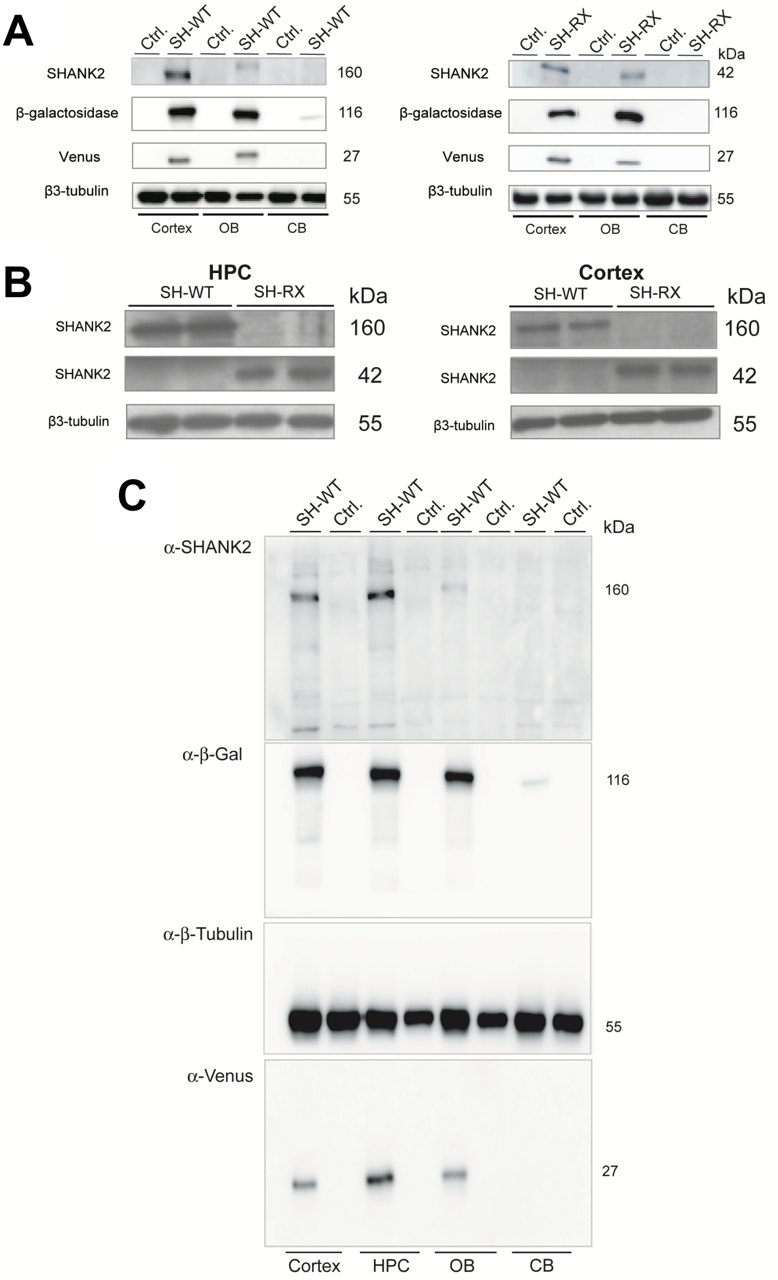

Fig. S2. Immunoblot expression analysis. A. Expression of the transgenic SHANK2A (left) and SHANK2A(R462X) (right) in the cortex, OB and CB of SH-WT and SH-RX mice, respectively. **B**. Expression of the transgenic SHANK2A and SHANK2A(R462X) in the hippocampus of two animals per genotype. The proteins were visualized by immunoblotting using antibodies specific to the human SHANK2, β-galactosidase and Venus. β3-tubulin was used as a loading control. SHANK2A (SH-WT); SHANK2A(R462X) (SH-RX). **C.** Validation of SHANK2, β-galactosidase, Venus and β3-Tubulin antibodies in different brain regions of SH-WT mice in immunoblots. Immunoreactive proteins on nitrocellulose membranes detected by human SHANK2, β-galactosidase, β3-tubulin, and Venus were visualized and recorded by the Fujifilm LAS-3000 in the sampling mode with 2 – 5 sec intervals. Images that were out of the linear recording mode were excluded from the quantitative analysis. HPC: Hippocampus, OB: Olfactory bulb, CB: Cerebellum.

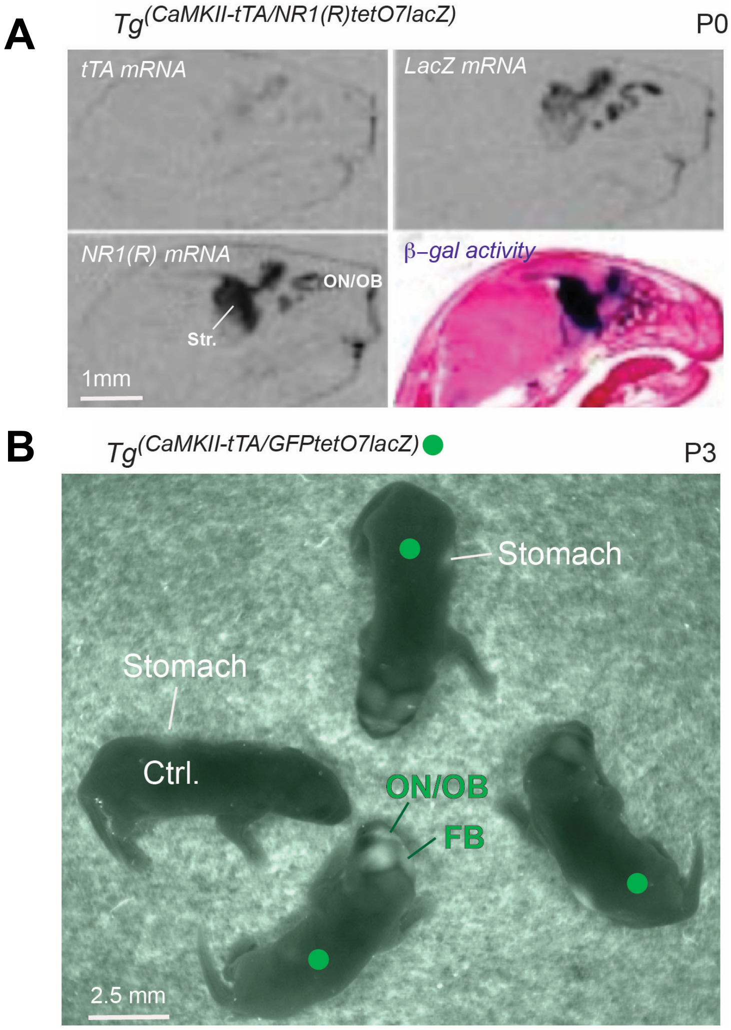

Fig. S3. **Prenatal and early postnatal expression of the *transgene TgαCaMKII-tTA* as monitored by tTA reporter transgenic expression units*.* A.** In situ hybridization (ISH) on sagittal sections of double transgenic $Tg^{Camk2a\text{-}tTA/NR1(R)tetO7lacZ}$ mice revealed the expression of tTA (top left), LacZ (top right) and NR1(R) (bottom left) in Str. and OB at P0. The expression was confirmed using X-gal staining to visualize β-galactosidase activity (bottom right). **B.** Double transgenic $Tg^{Camk2a\text{-}tTA/GFPtetO7lacZ}$ mice (indicated by the green spots) revealed an autofluorescence of GFP in the ON/OB and FB at P3. In this system, the milk in the stomach of the pups can be detected by a false positive signal (for details, see Krestel et al., 2001[1]. Str.: Striatum, ON: Olfactory nucleus, OB: Olfactory bulb, FB: Forebrain. The genetic mouse model used in **A** was described in detail in[1,2].

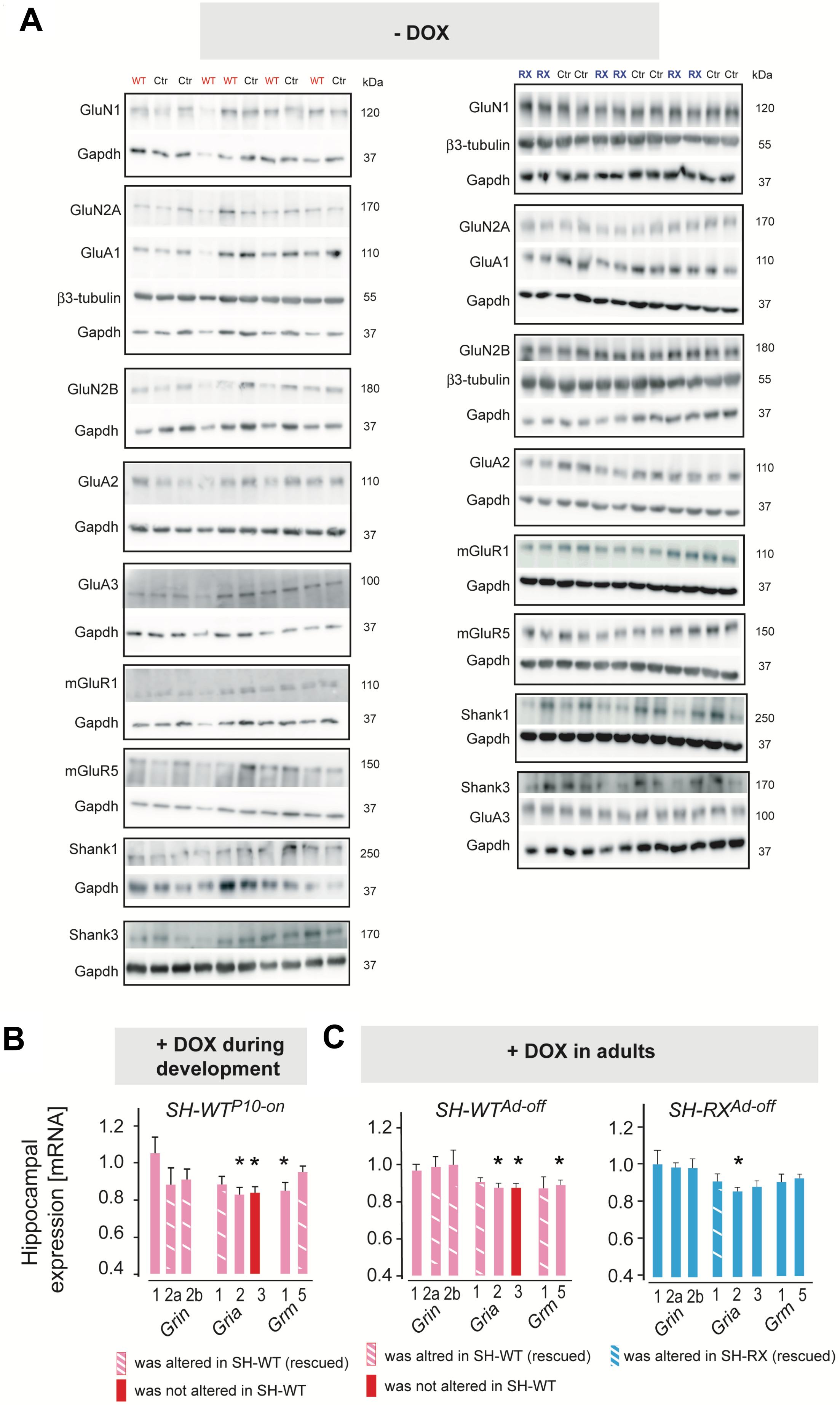
A
- DOX
WT Ctr Ctr WT WT Ctr WT Ctr WT Ctr
kDa
GluN1
Gapdh
GluN2A
GluA1
β3-tubulin
GluN2B
GluA2
GluA3
mGluR1
mGluR5
Shank1
Shank3
RX RX Ctr Ctr RX RX Ctr Ctr RX RX Ctr Ctr
B
+ DOX during development
C
+ DOX in adults
SH-WT$^{P10\text{-}on}$
SH-WT$^{Ad\text{-}off}$
SH-RX$^{Ad\text{-}off}$
Hippocampal expression [mRNA]
Grin
Gria
Grm
was altered in SH-WT (rescued)
was not altered in SH-WT
was altred in SH-WT (rescued)
was altered in SH-RX (rescued)

Fig. S4. Expression analysis of SHANK and glutamate receptors in SH-WT and SH-RX mice. A. Immunoblot analysis of hippocampal extracts from 5 SH-WT and 5 control littermates and 6 SH-RX and 6 control littermates. **B.** Expression analysis of SH-WT$^{P10\text{-}on}$ hippocampi by nCounter revealed a significant down-regulation of *Gria2*, *Gria3* and *Grm1* indicated by asterisks (n=7 SH-WT and 8 control mice, 3 – 5 months). **C.** Expression analysis of hippocampi by nCounter revealed a significant downregulation of the *Gria2,3 and Grm5* mRNA for SH-WT$^{Ad\text{-}off}$ mice. For SH-RX$^{Ad\text{-}off}$ mice, *Gria2* expression was downregulated (n = 7 SH-WT$^{Ad\text{-}off}$ and 8 Ctrl.$^{AD\text{-}off}$ mice at 5 – 8 months of age; n = 7 SH-RX$^{AD\text{-}off}$ and Ctrl.$^{AD\text{-}off}$ mice at 5 – 8 months of age). Unpaired two-tailed Student's t-test followed by the Benjamini-Hochberg test, *$p \leq 0.05$. Error bars indicate the standard error of the mean (SEM).

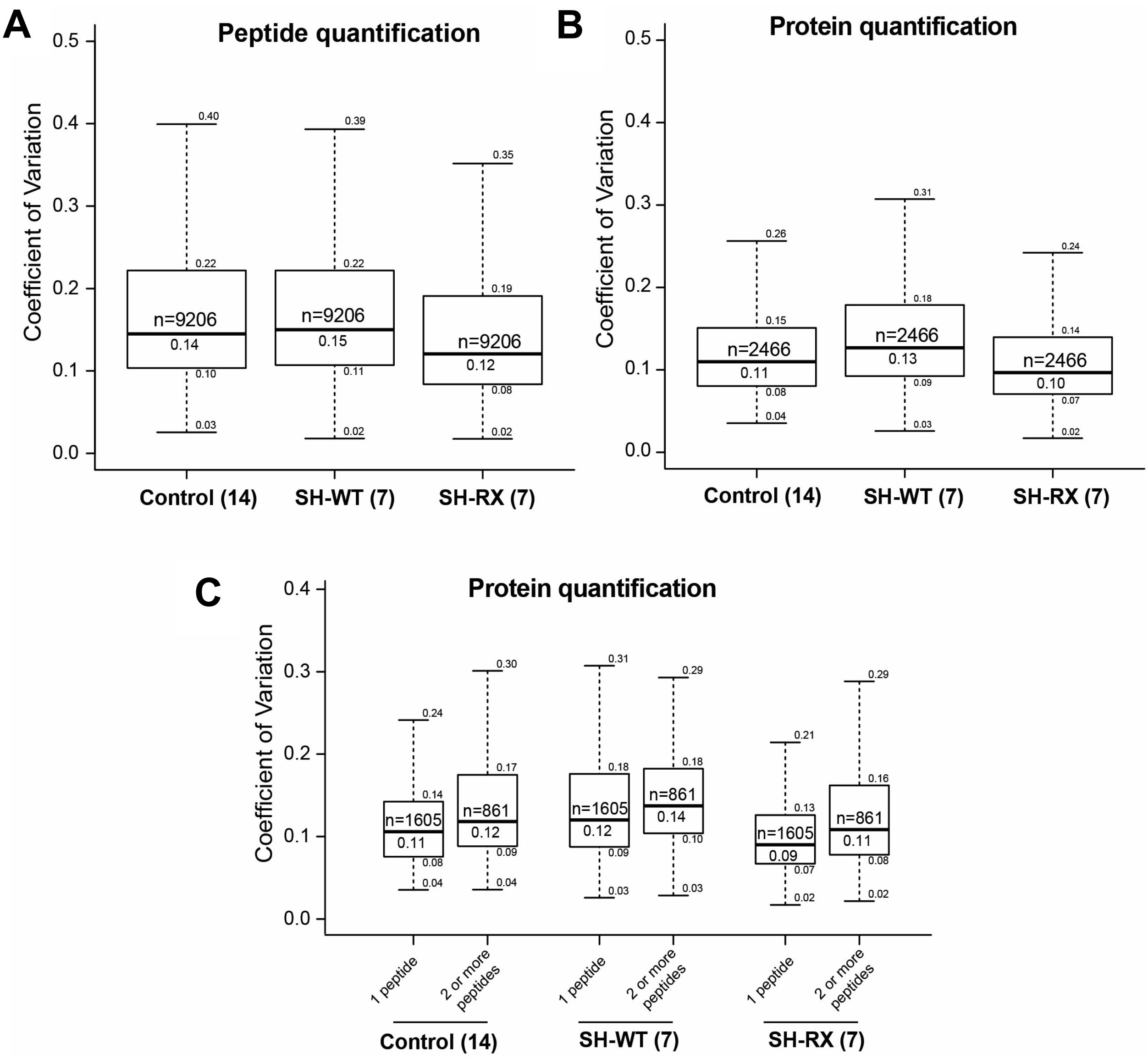

Fig. S5. Peptide and protein quality control for the SWATH analysis. Boxplots showing the coefficient of variation (CoV) at **A.** peptide level; **B.** protein level and **C.** protein level of the proteins quantified with one and with 2 or more peptides in each experimental condition, as a quality metric of the reproducibility of replicate measurements. The number (n) of peptides or proteins analyzed is given together with the median CoV (black line across the box). Replicas were for controls 14, for SH-WT 7 and for SH-RX 7 biologically independent samples (animals) of the synaptically enriched protein fraction. The box length indicates the interquartile range. e.g in (B) The control group showed 11% median CoV for the 2466 proteins quantified in 14 biological replicates.

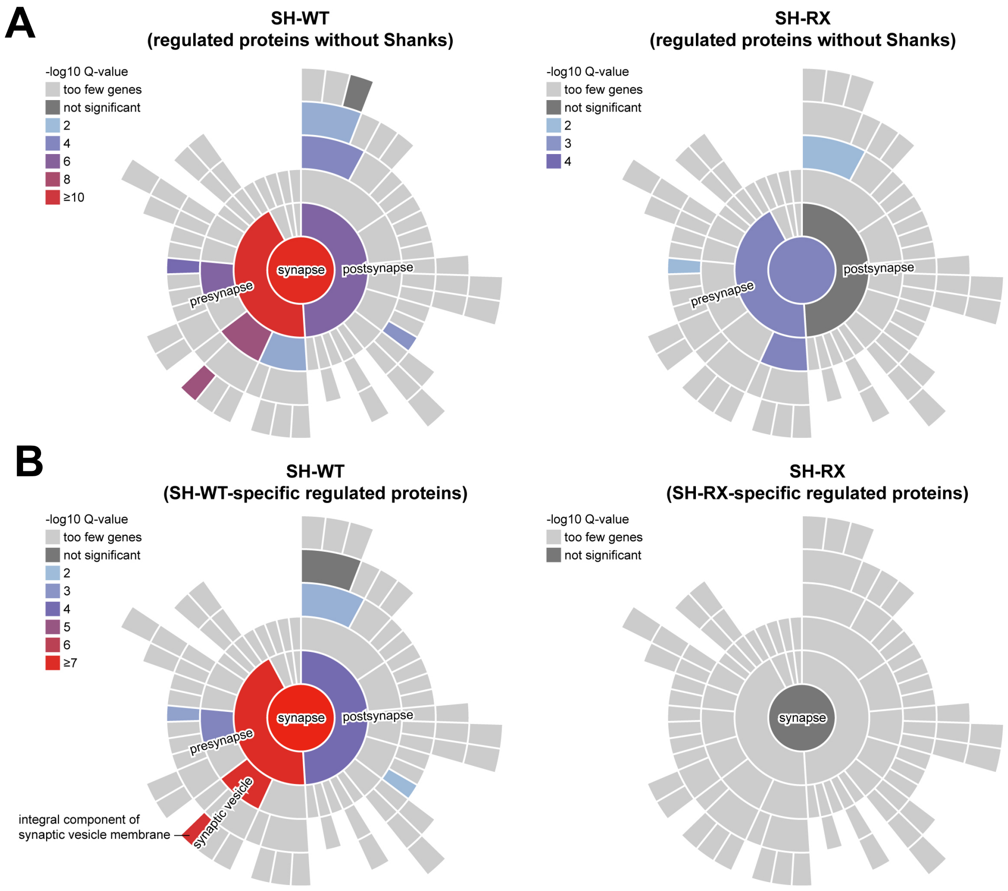

Fig. S6. SYNGO enrichment analysis A. Enrichment analysis of the significantly regulated proteins (excluding Shank proteins) using brain expressed genes as background identified the most prominent enrichment of altered proteins in the presynaptic component. **B.** Enrichment analysis of the proteins specifically regulated in SH-WT, strengthens further the prominent enrichment of altered proteins in the presynaptic versus postsynaptic proteins in both lines**.** Terms are color-coded according to enrichment Q-value.

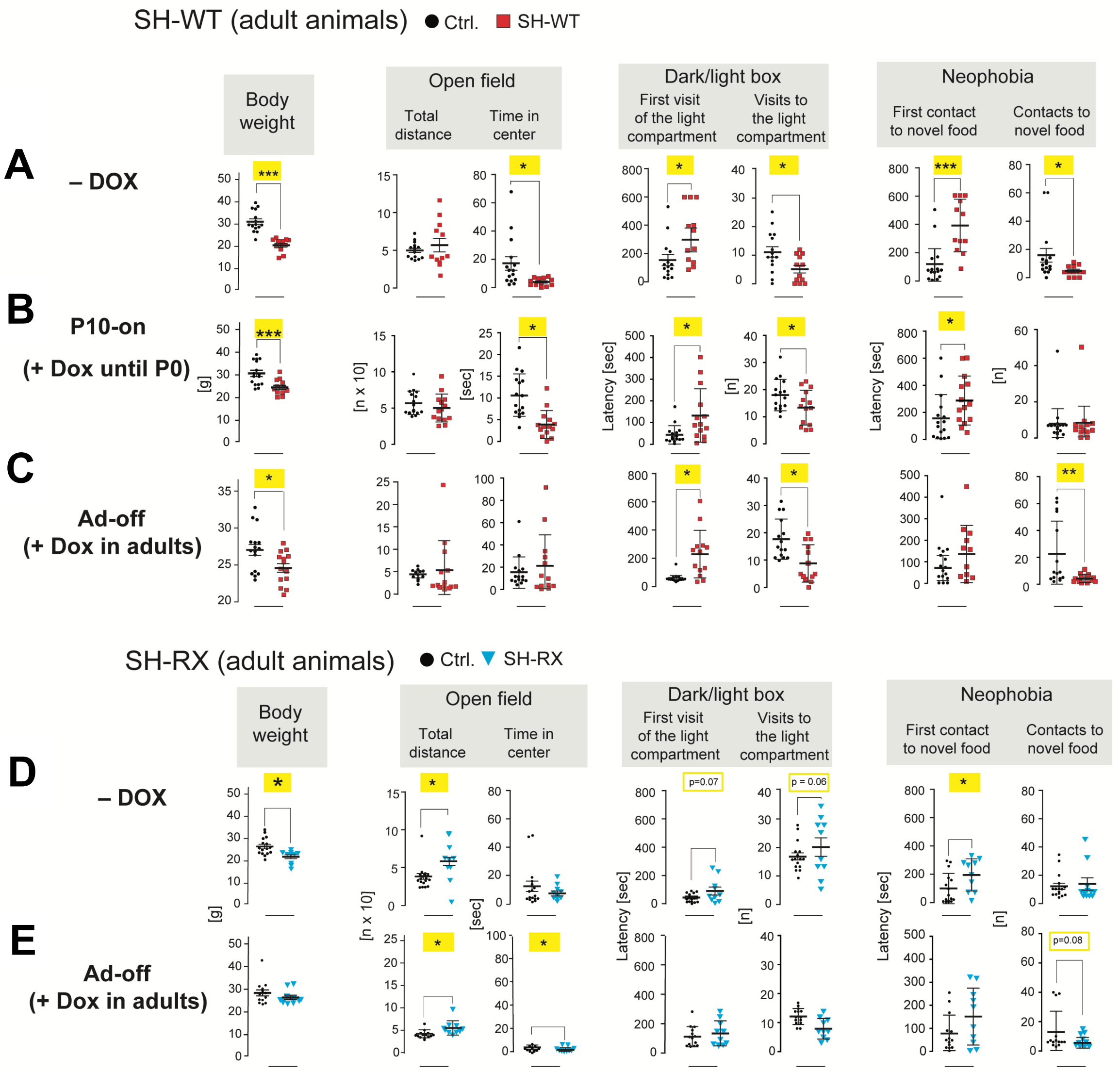

Fig. S7. Body weight and anxiety in mouse lines. Body weight of **A.** SH-WT, **B.** SH-WT$^{P10\text{-}on}$ and **C.** SH-WT$^{Ad\text{-}off}$ was 30%, 20% and 10% lower, respectively, compared to their respective control mice. Body weight of **D.** SH-RX, but not **E.** SH-RX$^{Ad\text{-}off}$ mice were 10% lower than their respective control. For anxiety analysis, **A.** SH-WT, **B.** SH-WT$^{P10\text{-}on}$, **C.** SH-WT$^{Ad\text{-}off}$, **D.** SH-RX and **E.** SH-RX$^{Ad\text{-}off}$ were analyzed in the open field, dark/light box and by the latency to explore or drink novel food. All mouse cohorts showed some sort of anxiety. However, anxiety was not consistently apparent in the three tests. In the 10 min exploration of the open field test, mild hyperactivity could only be detected in SH-RX mice.

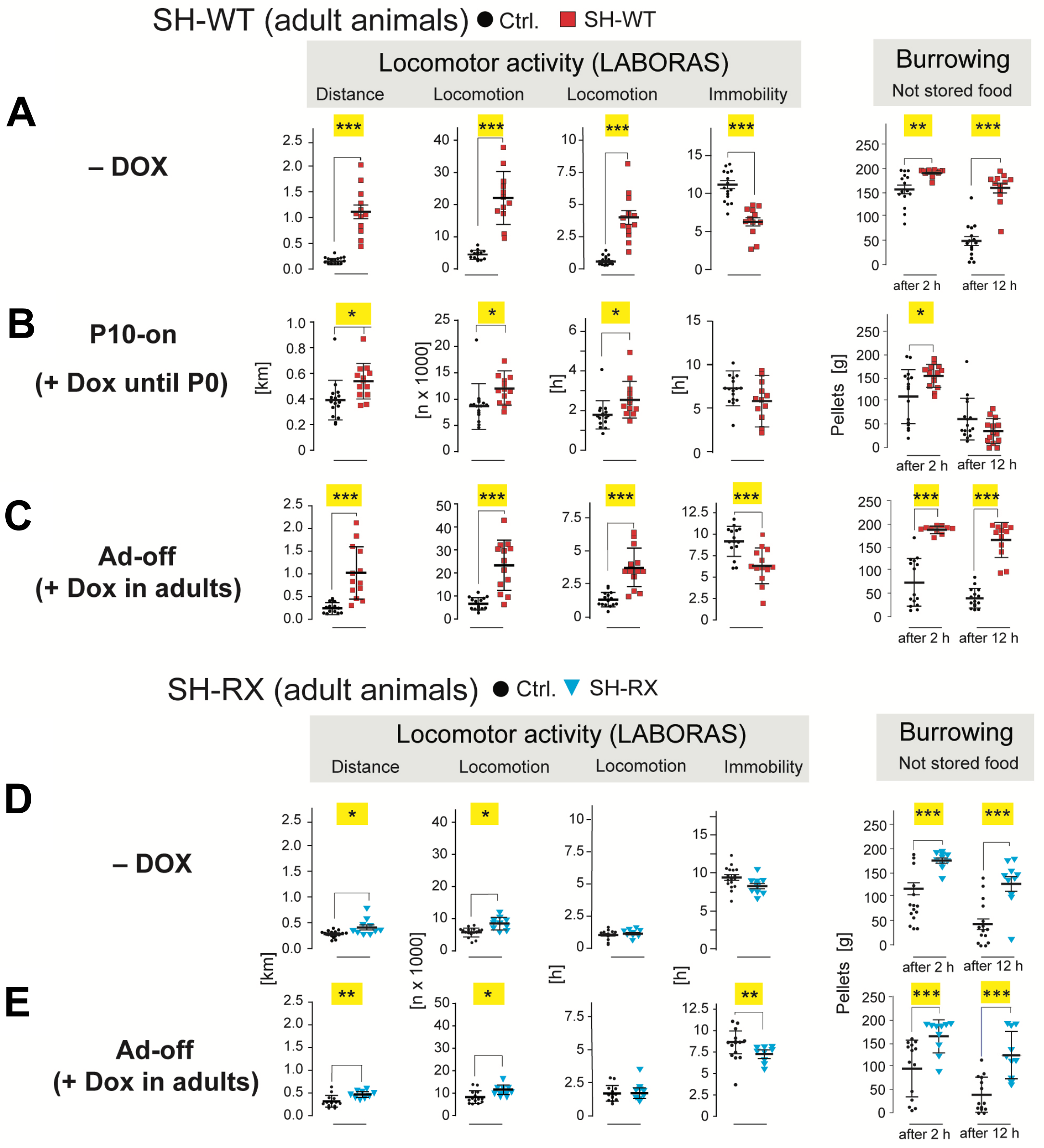

Fig. S8. Locomotion and burrowing test. Hyperactivity was observed in **A.** SH-WT, **B.** SH-WT$^{P10\text{-}on}$, **C.** SH-WT$^{Ad\text{-}off}$, **D.** SH-RX and **E.** SH-RX$^{Ad\text{-}off}$ mice with longer distance traveled, duration and number of locomotion and decreased immobility, but was more pronounced in SH-WT and SH-WT$^{Ad\text{-}off}$ mice. In the burrowing test, the amount of food left in a tube was significantly increased in all 5 cohorts after 2h compared to controls. After 12 h, only the SH-WT$^{P10\text{-}on}$ mice showed regular burrowing.

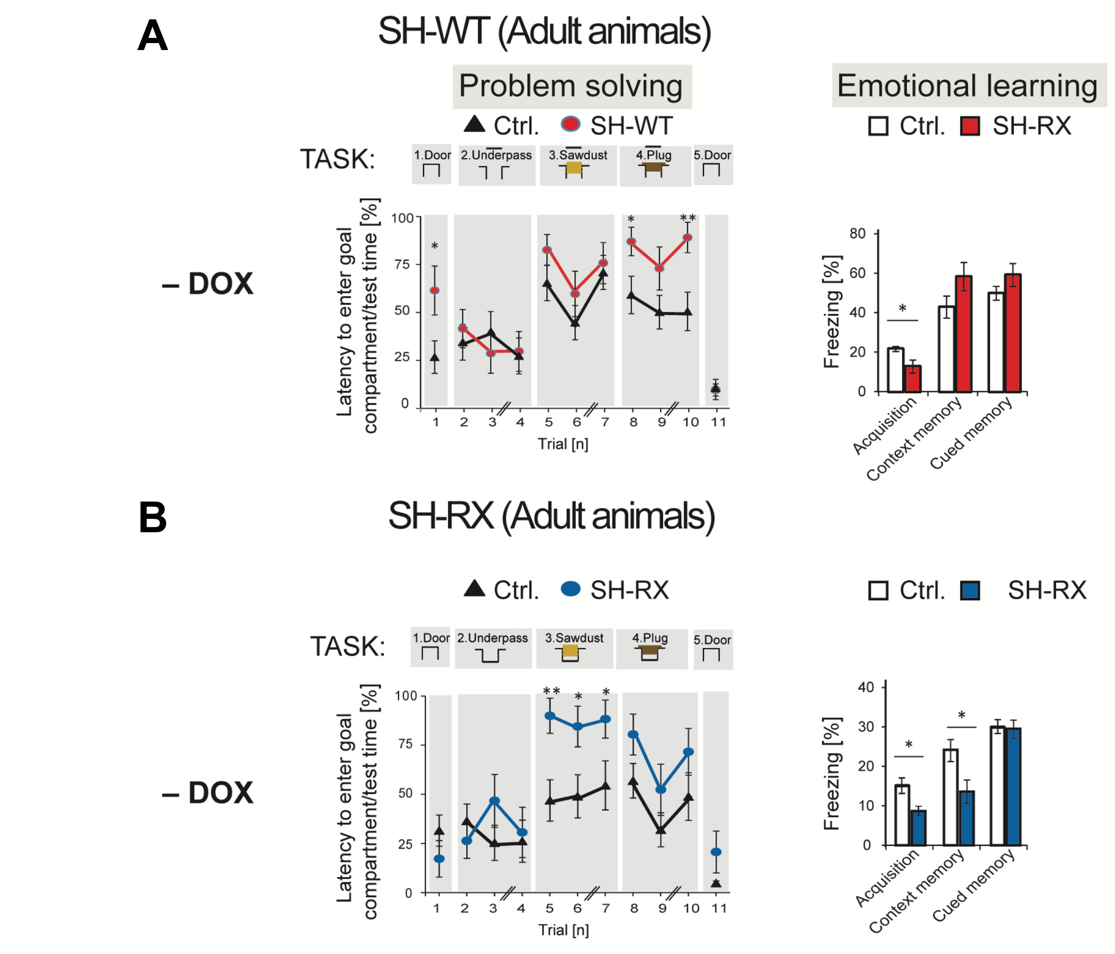

Fig. S9. Cognitive and emotional behavioral tests. A. In the puzzle box test, less ability to solve the puzzle was shown for SH-WT in sessions 1, 8 and 10 with no impairment in either the context or cued memory in the fear conditioning test (n=12 SH-WT and 15 littermate control mice, 6-8 months). **B.** SH-RX mice showed less ability to solve the puzzle in the sawdust trials (5, 6 and 7) in the puzzle box test with a reduced context memory in the fear conditioning test (n =10 SH-RX and 16 littermate control mice, 6-8 months).

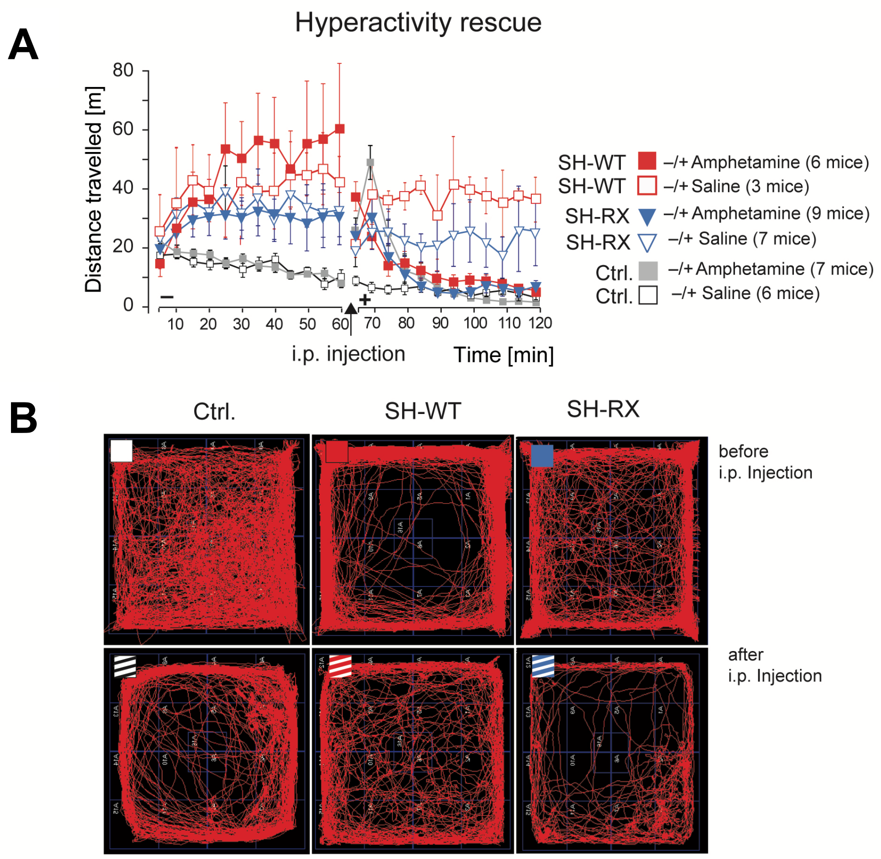

Fig. S10. Effect of amphetamine on SH-WT and SH-RX mice. A. In the open field, i.p. injection of amphetamine but not of saline attenuated the hyperactivity of SH-WT and SH-RX mice. **B.** In the trace maps, SH-WT and SH-RX travel distances were significantly less compared to the 60 min time window trace maps before amphetamine injection. Of note, the increased jumping activity in the four corners of the open field in SH-RX traces before amphetamine injection.

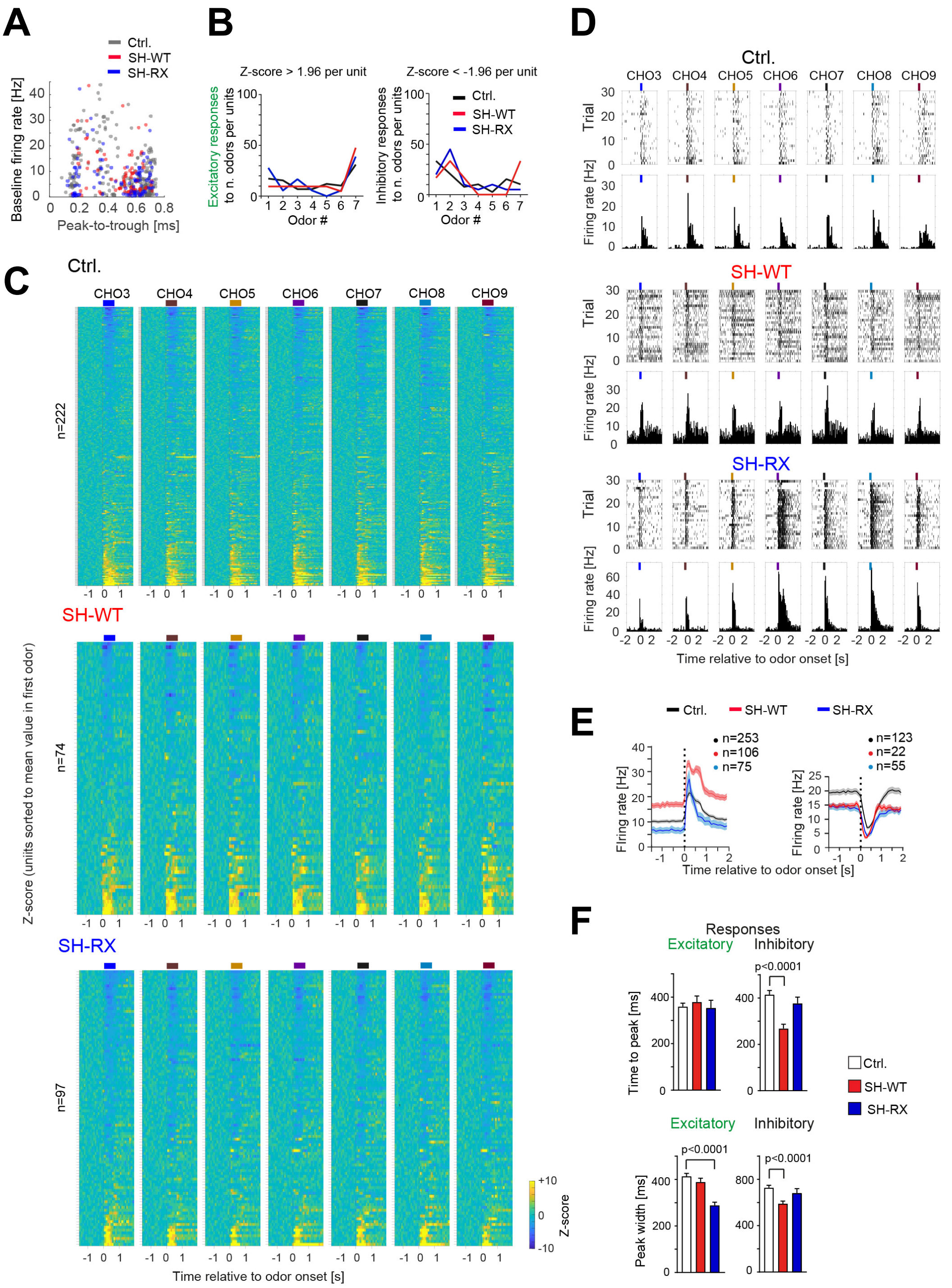

Fig. S11. Unit responses in the olfactory cortex of SH-RX, SH-WT and controls. A. plot of the mean spike width (from peak to trough) and the mean baseline firing rate for each unit in the three genotypes. **B.** The number of significant (left) excitatory and (right) inhibitory responses (-1.96>z-score >1.96) per unit, which showed any significant response was comparable across genotypes. **C.** Heat plots of the z-score of the firing rate of all units recorded in (top) control (middle) SH-WT and (bottom) SH-RX mice. Units were sorted according to their z-score in the response to the CHO3 odorant in a window of 1 s from inhalation onset. For all other odorants, the unit order of CHO3 was kept. **D.** Raster plot of spikes (upper part) and peri-stimulus histogram of the mean firing rate (lower part) of a unit from (top) control (middle) SH-WT and (bottom) SH-RX mice. For each genotype, units were chosen with broad responses to all odorants with increasing c-

chain length (CHO3-9) applied for 0.5 s. **E.** Mean firing rate (without baseline subtraction) of all cell-odor pairs with an excitatory or inhibitory response to the odorants for each genotype. **F. top.** Time to peak response from odor onset of all cell-odor pairs with an excitatory (ANOVA: $p=0.76$) or inhibitory (ANOVA: $p<0.0001$) odor response**; bottom.** and half-width of the odor response of all cell-odor pairs with an excitatory (ANOVA: $p<0.0001$) or inhibitory (ANOVA: $P=0.003$) odor response for the three genotypes. Only significant posttests are indicated in the figure bar graphs.

Table S1: Alteration in synapse-enriched protein levels from the hippocampi of SH-WT and SH-RX mice. Mutations in genes that are highlighted in red are associated with neuropsychiatric or neurodegenerative disorders. Genes that are highlighted in green are also differentially expressed in Camk2a-tTA mice. Differently expressed genes related to postsynaptic functions are highlighted in yellow. Proteins that were found in SYNGO, and which were annotated in SYNGO are labeled by (++). Syngoportal.Org (Gene list using "ID convert tool" followed by "start gene set analysis" and finally by "annotations". P values ≤ 0.05 are highlighted in gray.

Gene	# Peptides	SH-WT vs controls		SH-RX vs controls		Function/localization in neurons
		Fold-change	FDR adjusted p-values	Fold-change	FDR adjusted p-values	
Kif1a[3]	7	2.18	1.14E-09	1.83	4.63E-09	Axonal transport
Eva1a	1	1.55	2.18E-07	0.98	0.7665	Neuronal differentiation
Rae1	1	1.51	4.16E-07	1.41	0.0003	Synapse formation
Shank3	8	0.66	4.16E-07	0.95	0.4375	++ Postsynapse
Pgls	1	1.60	6.77E-07	1.19	0.0102	Metabolic enzyme
Mycbp2[4]	1	1.76	1.92E-05	1.61	0.0007	Both
Map7d2	1	1.39	6.17E-05	1.24	0.0279	Proximal axon
Shank2/SHANK2A	13	2.39	7.34E-05	0.87	0.5450	++ Postsynapse
Ndrg3	4	1.24	0.0001	1.24	3.41E-05	Gener. of new neurons
Apba2[5]	2	1.32	0.0001	1.22	0.0005	Vesicle release
Shank1	14	0.74	0.0003	0.97	0.7490	++ Postsynapse
C1qc	2	0.67	0.0003	1.09	0.4087	Pruning of synapse
Slc6a7	4	0.79	0.0005	0.87	0.0987	++ Presynapse
Serpinb1a	1	0.56	0.0008	0.73	0.0136	Neurotrophic
Cadm3	4	0.76	0.0013	0.82	0.0342	++ Contact axon-glia
Rgs14	3	0.71	0.0014	0.72	0.0022	Both, G-protein regulator
Cd44	1	0.79	0.0016	0.96	0.6913	Both
Homer2[6]	1	1.30	0.0019	0.99	0.9010	++ Postsynapse
Mapk10	1	1.17	0.0029	1.09	0.0681	++ Postsynapse
Rasgrp1	1	0.78	0.0031	0.87	0.1001	Parkinson
Nptxr	2	0.82	0.0032	0.97	0.6410	Postsynapse
Grm3	7	0.73	0.0035	0.88	0.2845	++ Both
Cyb5r1	4	0.78	0.0045	0.91	0.3434	Membranes of synapses
Dkk3	1	0.81	0.0057	0.88	0.0790	Neuroprotection
Pgm2l1	7	1.48	0.0062	1.51	0.0022	Metabolic enzyme
Stxbp6[7]	2	0.63	0.0077	0.68	0.0330	Binds t-Snare
Slc18a2 /VMAT2	1	0.85	0.0079	0.93	0.2111	++ Presynaptic vesicles
Snph	2	0.82	0.0098	0.93	0.3092	Docking axonal mitochondria
Idh2	7	1.25	0.0098	1.01	0.9234	Neuronal Apoptosis
Slc25a22	2	0.79	0.0104	0.94	0.4809	Neuronal Mitochondria
Sccpdh	1	0.83	0.0104	1.02	0.7705	
Epha4	6	0.83	0.0105	0.87	0.0136	++ Both
Rasal1	8	0.83	0.0125	0.96	0.5676	
Snx27	3	1.20	0.0126	1.11	0.1479	
Slc16a1	1	0.75	0.0126	0.99	0.9538	
Ptprn2[8]	1	0.62	0.0133	0.63	0.0301	++ Synaptic vesicle Pro.
Rmnd1	3	0.83	0.0133	0.92	0.1530	
Nwd2	6	0.80	0.0133	0.78	0.0136	
S100a13	2	0.81	0.0133	0.93	0.4032	
Tmem132a	1	0.75	0.0136	0.83	0.1573	
Fam171a2	2	0.81	0.0136	0.97	0.7397	
Cntnap1[9]	12	0.86	0.0140	0.87	0.0415	++ Presynapse
Traf3	1	0.65	0.0148	0.98	0.8337	
2010300c02rik	4	1.39	0.0154	1.08	0.6367	
Rac3	1	0.82	0.0154	0.94	0.4809	

Stxbp5	5	0.82	0.0169	0.94	0.4868	++ Presynapse
Vti1a	1	0.84	0.0170	0.97	0.6489	++ Presynapse
Rab15	1	0.51	0.0170	1.05	0.7714	
Ptprs	9	0.82	0.0173	0.89	0.1001	++ Presynapse
Dgkb	2	0.79	0.0180	0.90	0.3057	++ Postsynapse
Atp8a1	14	0.86	0.0181	0.93	0.3421	++ Presynapse
Gltp	1	0.79	0.0195	0.86	0.1700	
Lman1	1	1.19	0.0199	1.00	0.9873	
Baiap2[22]	12	0.86	0.0203	0.94	0.3932	++ Both
Abhd3	1	0.78	0.0203	0.92	0.4542	
Scamp4	1	0.81	0.0203	0.99	0.9254	
Stx16	1	0.82	0.0215	0.94	0.5026	++ Presynapse
Pip4k2c	4	1.37	0.0215	1.11	0.5325	
Rimbp2[10/19]	3	0.86	0.0244	0.83	0.0136	++ Presynapse
Syt17	1	0.71	0.0244	0.74	0.1001	
Mlf2	1	0.80	0.0246	0.87	0.1352	
Cacng8	3	0.83	0.0246	1.00	0.9697	++ Postsynapse
Gria1[11]	11	0.81	0.0262	0.82	0.0294	++ Postsynapse
2310061i04rik	1	0.79	0.0277	0.92	0.2981	
Nos1[12]	2	0.84	0.0282	0.88	0.0296	++ Postsynapse
Them4	4	0.88	0.0287	0.92	0.0904	
Unc5c	1	0.73	0.0291	0.83	0.1611	
Ppap2b	4	0.82	0.0291	0.94	0.4399	
Coro2b	3	0.82	0.0292	0.92	0.4154	
Calu	3	1.22	0.0295	1.18	0.1716	
Itm2b	1	0.49	0.0295	1.02	0.9538	
Myadm	1	0.89	0.0298	0.95	0.4052	
Rtn4ip1[19]	1	0.79	0.0299	0.79	0.0342	Mitochondria
Cpne5[13,14]	4	0.83	0.0304	0.82	0.0415	Neurite outgrowth
Cpne6	8	0.82	0.0304	0.82	0.0498	++ Postsynapse
Smpd3	1	0.78	0.0304	1.02	0.9199	
Kcnn2	2	0.83	0.0311	0.92	0.3454	++ Postsynapse
Rab11fip2	2	0.84	0.0317	0.91	0.3434	
Il1rap	1	0.83	0.0321	0.87	0.1001	
Plxna1	8	0.83	0.0327	0.80	0.0277	Axon guidance
Synpr	2	0.78	0.0327	0.84	0.2184	++ Synaptic vesicle protein
Rmdn3	3	0.81	0.0327	0.98	0.8659	++ Both
Rpl13a	1	1.25	0.0371	0.91	0.3155	++ Postsynapse, Ribosomome
Bsg	4	0.87	0.0372	0.90	0.1144	
Atp6v0d1	5	0.86	0.0372	0.98	0.8508	++ Synaptic vesicle
Plxna2	3	0.76	0.0424	0.79	0.1001	Axon guidance
Txnl1	1	1.32	0.0424	1.14	0.1827	
Pycrl	1	0.81	0.0424	1.05	0.6754	
Gm996	4	0.81	0.0438	0.95	0.5439	
Samm50	6	0.86	0.0438	0.97	0.7049	++ Presynapse
Svop	1	0.74	0.0443	0.96	0.7871	++ Presynapse
Gng7	1	0.81	0.0464	0.83	0.0819	
Myo18a	6	1.46	0.0464	1.15	0.2845	
E9Q6C7	1	0.79	0.0464	0.90	0.3956	
Sept2	2	0.89	0.0479	0.99	0.9086	
Syt12	2	0.85	0.0485	0.94	0.4154	++ Presynapse
Mark4	1	0.86	0.0485	0.96	0.6549	
Vdac1	6	0.88	0.0485	0.98	0.7490	++ Both
Mtch2	3	0.82	0.0485	0.98	0.8166	
Slc6a11	3	0.76	0.0485	1.03	0.8608	++ Both
Ipo5	5	1.18	0.0486	1.03	0.6489	
Map1lc3b	1	0.89	0.0488	0.97	0.6369	
Pdcl	1	0.84	0.0489	0.96	0.5938	

Pmm1	2	1.36	0.0489	1.03	0.8221	
Ca4	2	2.42	0.0585	3.11	0.0277	
Plxna4	20	0.87	0.0617	0.84	0.0321	Axon guidance
Lancl1	3	1.14	0.0808	1.17	0.0136	
Cryz	2	0.90	0.0962	0.85	0.0368	
Cpne4	3	0.90	0.0970	0.82	0.0028	
Slc7a14	4	0.86	0.1059	0.84	0.0489	
Pde1a	5	1.20	0.1296	1.25	0.0454	
Hsd17b8	2	0.92	0.1500	0.87	0.0446	
Slc35f1	1	1.29	0.1928	1.55	0.0406	
Lxn	2	1.16	0.1998	1.28	0.0294	
Ccdc177	1	0.93	0.2111	0.88	0.0375	
Gpr56	1	1.14	0.2205	1.26	0.0406	
Uchl1	3	1.18	0.2270	1.32	0.0342	
Ermp1	2	0.93	0.2414	0.84	0.0084	
Kit	2	0.87	0.2476	0.70	0.0321	
Ech1	1	1.13	0.2488	0.79	0.0294	
Pld3	3	1.07	0.2764	1.15	0.0279	
Anxa11	4	0.91	0.3161	0.76	0.0224	
Scn8a	3	0.91	0.3885	0.86	0.0303	++ Both
Gm20671;Pisd	2	0.95	0.3937	0.79	0.0415	
Cpne7	5	0.90	0.4758	0.75	0.0415	
Ppm1f	1	0.97	0.6217	0.87	0.0303	
Pgbd5	1	0.96	0.6378	0.86	0.0406	
6330403a02rik	2	0.96	0.6446	0.86	0.0415	
Pex11b	1	0.97	0.6508	0.87	0.0321	
Tnks1bp1	2	0.97	0.7521	0.86	0.0489	
Rhoa;Rhoc	2	0.97	0.7753	1.22	0.0303	++ Postsynpse
Wbscr17	3	1.01	0.9113	0.83	0.0330	
Vps16	3	1.01	0.9408	1.10	0.0489	++ Presynapse
Acot13	2	1.00	0.9649	0.86	0.0303	
Them6	1	1.00	0.9654	0.82	0.0415	
Clcn6	3	1.00	0.9949	1.13	0.0337	

Genes differentially expressed
Genes associated with psychiatric disorders
Genes differentially expressed in Camk2a-tTA mice (KT1) see **Table S5**
Postsynaptic localization

Table S2: Differentially expressed **hippocampal genes in SH-RX mice.** Mutations in genes are as in Table S3 but now sorted according to FDR adjusted P values. Differentially expressed genes related to postsynaptic functions are highlighted in yellow (see Table S3). For color code in the first column, see Table S3.

Gene	SH-RX vs controls fold change	SH-RX vs controls FDR adjusted p-values	Function/localization
Kif1a[3]	1.83	4.63E-09	Axonal transport
Ndrg3	1.24	3.41E-05	Nuclear; Cell growth
Rae1	1.41	0.000274634	Synapse formation
Apba2[5]	1.22	0.000499013	Axon trafficking
Mycbp2[4]	1.61	0.000722405	Both
Pgm2l1	1.51	0.002230564	Metabolic enzyme
Rgs14	0.72	0.002230564	Both
Cpne4	0.82	0.002830756	Outgrowth of processes
Ermp1	0.84	0.008358106	ER
Pgls	1.19	0.010176294	Metabolic enzyme
Lancl1	1.17	0.013645122	Neuron survival
Rimbp2	0.83	0.013645122	Presynapse
Epha4	0.87	0.013645122	Both
Nwd2	0.78	0.013645122	Both/actin regulation
Serpinb1a	0.73	0.013645122	Neurotrophic
Anxa11	0.76	0.022434897	Ca^{2+} Homeostasis
Ca4	3.11	0.027671	Carbonic anhydrase
Plxna1	0.80	0.027671	Axon guidance
Map7d2	1.24	0.027864551	Proximal axon
Pld3	1.15	0.027864551	Phospholipase D
Lxn	1.28	0.029387066	Tumor suppressor
Ech1	0.79	0.029387066	Soma, Dendrites
Gria1[11]	0.82	0.029387066	Postsynapse/membrane
Nos1[12]	0.88	0.029595978	Postsynapse
Ptprn2[8]	0.63	0.030111267	Vesicle secretion
Scn8a	0.86	0.030268813	Node of Ranvier
Acot13	0.86	0.030289017	Acyl-CoA hydrolysis
Rhoa;rhoc	1.22	0.030289017	Presynapse
Ppm1f	0.87	0.030289017	Cell stress buffer
Pex11b	0.87	0.032101342	Neuronal migration
Kit	0.70	0.032101342	Growth factor receptor
Plxna4	0.84	0.032101342	Axon guidance
Wbscr17	0.83	0.033011016	Lamellipodium formation
Stxbp6[7]	0.68	0.033011016	Transporter in vesicles
Clcn6	1.13	0.033737627	Lysosomal storage
Uchl1	1.32	0.034195896	Neuronal injury
Rtn4ip1	0.79	0.034195896	Dendritic growth
Cadm3	0.82	0.034195896	Contact axon-glia
Cryz	0.85	0.036849927	Quinone oxidoreductase
Ccdc177	0.88	0.037525782	Coiled-coiled domain protein
Slc35f1	1.55	0.040580386	Transmembrane transporter
Gpr56	1.26	0.040580386	Neuronal migration
Pgbd5	0.86	0.040580386	Transposon protein
Them6	0.82	0.041542566	Thioesterase
6330403a02rik	0.86	0.041542566	Neuronal marker
Gm20671;pisd	0.79	0.041542566	Node of Ranvier
Cpne7	0.75	0.041542566	Cell differentiation
Cntnap1[9]	0.87	0.041542566	Presynapse

Cpne5[13,14]	0.82	0.041542566	Neurite outgrowth
Hsd17b8	0.87	0.044621876	Mitochondria
Pde1a	1.25	0.04541138	Cyc. Nuc. Phosphodiest.
Vps16	1.10	0.048906198	Presynaptic
Tnks1bp1	0.86	0.048906198	Tankyrase-1-bind. protein
Slc7a14	0.84	0.048906198	Lysosomal localized
Cpne6	0.82	0.049821894	Ca^{2+} sensor

Genes differentially expressed in SH-WT and SH-RX (see **Table S3**).
For the color codes in the first column see **Table S3**.

Table S3: Alteration in synapse-enriched protein levels from the hippocampi of transgenic KT1 (*Tg*$^{Camk2a\text{-}tTA}$) and wild-type control mice. Genes highlighted in green were listed in Table S3 in SH-WT and/or SH-RX mice as differentially expressed. Cut off of FDR adjusted P values ≤ 0.05.

Gene	Fold change	p-value	FDR adjusted p-values
Stxbp6[7]	0.584111467	1.4287845E-09	3.4590872E-06
Ca4[15]	4.009301583	1.0676870E-08	1.2924352E-05
Slc35f1	1.857593191	2.7592248E-08	2.2266944E-05
Olfm1	0.766127951	1.1867941E-06	0.0007183
Scg3	0.762382231	2.9637421E-06	0.0014350
Cpne7	0.703610436	1.7631503E-05	0.0071143
Aldh1b1	1.353040824	2.6867273E-05	0.0092922
Ptprn2[8]	0.595345824	3.1996665E-05	0.0096830
Adgrb2	0.711381752	4.6484992E-05	0.0125045
Cpne5[13,14]	0.802226982	6.2159861E-05	0.0150489
Tpbg	0.666049461	7.1904545E-05	0.0158255
Mal2	0.834569888	9.6085728E-05	0.0178941
Slc2a12	0.808782733	9.0513329E-05	0.0178941
Lrrtm1	0.784899168	0.0001084	0.0187436
Lxn	1.301927373	0.0001180	0.0190526
Cdh13	0.681560583	0.0001728	0.0246026
Mtnd2	3.511112672	0.0001694	0.0246026
Gabrb2	0.819076622	0.0002022	0.0271969
Gprin1	0.755129595	0.0002276	0.0279656
Mlf2	0.815984152	0.0002311	0.0279656
Tpp1	0.826847404	0.0002426	0.0279656
Ntrk3	0.800467473	0.0002704	0.0297581
Gabra2	0.736327141	0.0003559	0.0309522
Gas7	0.821378174	0.0003350	0.0309522
Gng7	0.805143903	0.0003580	0.0309522
Ngef	0.764237944	0.0003540	0.0309522
Rap2b	0.821467914	0.0003227	0.0309522
Tfrc	0.833245843	0.0003193	0.0309522
Alcam	0.768304076	0.0003758	0.0313715
Rgs12	0.813187957	0.0004152	0.0335085
Lypd1	0.692205352	0.0004321	0.0337439
Ptprg	0.762074305	0.0004474	0.0338506
Negr1	0.778066008	0.0004665	0.0342211
Gm20503;Gng10	1.230576882	0.0005899	0.0420013
Pfn1	1.419474624	0.0006345	0.0436435
Rras2	0.82012858	0.0006670	0.0436435
Vgf	0.818633406	0.0006564	0.0436435
Kit	0.733114719	0.0007311	0.0462119
Rac3	0.837069453	0.0007444	0.0462119
Anxa11	0.778899913	0.0008429	0.0467966
Cpne2	0.753871507	0.0008636	0.0467966
Fam81A	0.775109678	0.0008698	0.0467966

Gabbr1	0.858401519	0.0007791	0.0467966
Prdx6	1.261447637	0.0008282	0.0467966
Rab3c	0.844168945	0.0008140	0.0467966
Synpo	0.830685523	0.0009039	0.0475735
Ocrl	0.784630039	0.0009453	0.0486949

Protein functions:

Stxbp6 encodes AMYSIN and is involved in the secretion of large dense-core vesicles by interacting with Syntaxin[7].

Ca4 encodes the carbonic anhydrase CA4. A dominant-negative point mutation in *Ca4* is involved in Retinitis pigmentosa.

Slc35f1 encodes the drug, metabolite transmembrane transporter called 'solute carrier family 35 member F1'. The function is unknown.

Ptprn2 encodes the receptor type tyrosine-protease N2[8] resides within the deletion of the Camk2a-tTA transgene insertion site in Chr18[16]. Ptprn2 shows the highest expression in the brain *(https://www.ncbi.nlm.nih.gov/gene/?term=Ptpn2)* and is heterozygous in SH-WT, SH-RX and KT1 mice

Cpne5 encodes CopinV, which is Ca^{2+}-dependent membrane-binding protein. *Cpne5* is involved in anxiety, alcohol dependence and obesity[13,14].

Lnx encodes the E3 ubiquitin-protein ligase LNX1.

MLF encodes the myeloid leukemia factor 2.

Gng7 encodes the guanine nucleotide-binding protein subunit gamma.

Table S4: Antibodies for immunohistochemistry and western blot

Antibody	Host species	Concentration used	Reference number	Company	RRID
monoclonal anti-GFP	mouse	1 : 10000	ab38689	Abcam	AB_732715
monoclonal anti-Shank2	mouse	1 : 1000	75-088	Neuromab	AB_2254586
anti-β-galactosidase	mouse	1 : 20000	Z3783	Promega	AB_430878
monoclonal anti-β3-tubulin	mouse	1 : 20000	G7121	Promega	AB_430874
anti-mGluR1	rabbit	1 : 200	PA1-4516	Thermo fischer	AB_2294910
anti-mGluR5	mouse	1 : 1000	MABN540	Merck Millipore	not available
anti-GluN2A	rabbit	1 : 1000	07-632	Merck Millipore	AB_11213002
anti-GluN2B	rabbit	1 : 1000	NB300-106	Novus Biological	AB_10000537
anti-GluA1	rabbit	1 : 1000	AB1768-25UG	Chemicon	AB_2113602
anti-GluA2	rabbit	1 : 1000	AB1504	Chemicon	AB_2247874
anti-GluA3	rabbit	1 : 1000	ab40845	Abcam	AB_776310
anti-GluN1	rabbit	1 : 1000	AB9864R	Millipore „Merck“	AB_10807557
anti-Shank1	rabbit	1 : 1000	162013	Synaptic system	AB_2619859
anti-Shank3	mouse	1 : 1000	ab93607	Abcam	AB_10563849

Table S5: Oligonucleotide sequences of the nCounter probes for selected genes in SH-WT, SH-RX and control mice

Gene	Probe	Sequence
Gapdh	A	ATGGGCTTCCCGTTGATGACAAGCTTCCCATTCTCCCTCAAGACCTAAGCGACAGCGTGACCTTGTTTCA
	B	CGAAAGCCATGACCTCCGATCACTCATTTGATGTTAGTGGGGTCTCGCTCCTGGAAGATGGTG
Hspd1	A	CACAGTTCTTCCCTTTGGCCCCATTGTAACAGCTACAGCATCTGCTAAAACATCCTCTTCTTTTCTTGGTGTTGAGAAGATGCTC
	B	CGAAAGCCATGACCTCCGATCACTCGTGACCCCATCTTTTGTTACTTTGGGACTTCCCCAACTCTGTTCAATAAT
Sdha	A	GTAATCATACTCATCGACCCGCACTTTGTAATCTTCCCTGGCATGGGCTCCACAATTCTGCGGGTTAGCAGGAAGGTTAGGGAAC
	B	CGAAAGCCATGACCTCCGATCACTCCAGTGTTCCCCAAACGGCTTCTTCTGCTGTCCCTGGATGGGCTTGGA
Hprt1	A	CTCCGGAAAGCAGTGAGGTAAGCCCAACGCTCTCCCTGTTGAGATTATTGAGCTTCATCATGACCAGAAG
	B	CGAAAGCCATGACCTCCGATCACTCCAAAAAGCGGTCTGAGGAGGAAGCCGGCGGAGGAGGTGCTACCG
Pgk1	A	GAGGAGAACAGCGCGGCAGACGTGCGCTTTTGAAGCGTGCAGAATGCCAAAGACGCCTATCTTCCAGTTTGATCGGGAAACT
	B	CGAAAGCCATGACCTCCGATCACTCAAGCGACATTTTGGCAACACCGTGAGGTCGAAAGGCCCGGAGATGAGGAA
Gpi1	A	GGCCCCCAGAATGAAGGGTGTCAGCTTGGTAAACACAATAGAGTTGGTCGCGAACCTAACTCCTCGCTACATTCCTATTGTTTTC
	B	CGAAAGCCATGACCTCCGATCACTCTCCCACATGATGCCCTGAACAAAGATCTTGTGCTCATACATGGCAATCAA
Shank1	A	GAAGGCCGGTCATAACTTGGCTGGGAGCGGTGGTGAGGATCAAAGCTCGACTTTCGGGTTATATCTATCATTTACTTGACACCCT
	B	CGAAAGCCATGACCTCCGATCACTCCGATAGATTTCTGCCGGAGCATAAGGCCAGGTCCGGGAGGCAGGAAA
Shank2	A	GCAGAAATAAAGACAGCAAGTTAAGCAAGCAGGGCACGATCCTGTCCACACAACAGCCACTTTTTTTCCAAATTTTGCAAGAGCC
	B	CGAAAGCCATGACCTCCGATCACTCAACTCCGTGTGACTTCATTTTGGCCACCCTGGAATTATGCTAATCTGTTT
Shank3	A	CTTCGTCCGGTCCTCTCTGGTTTCATGCCGGGTGTCATACTGTCGCACCGTGTGGACGGCAACTCAGAGATAACGCATAT
	B	CGAAAGCCATGACCTCCGATCACTCTGTGAAGTGAGGCTGTCATAGGAACCCACAGTGTAGTGGCGGAAGAGACG
Venus	A	GGCTGTTGTAGTTGTACTCCAGCTTGTGCCCCAGGATGTTGCCTGGAGTTTATGTATTGCCAACGAGTTTGTCTTT
	B	CGAAAGCCATGACCTCCGATCACTCGTTGGCCTTGATGCCGTTCTTCTGCTTGTCGGCGGTGATATAGACGTTGT
Grm5	A	TGAGAGTGTCATATTTGGGAGAGGATGGGATGCAGAGGGCATAAAATTGGTTTTGCCTTTCAGCAATTCAACTT
	B	CGAAAGCCATGACCTCCGATCACTCGTTTCCAGTGGCTCACAACGATGAAGAACTCTGCGTGTAATCTCTGATGA
Grin1	A	CGTAGACCTGGCTAGAGATGAGGTCCTCACACACTGACAGCCAGCAGACCTGCAATATCAAAGTTATAAGCGCGT
	B	CGAAAGCCATGACCTCCGATCACTCAGTGAAGTGGTCGTTGGGAGTAGGCGGGTGACTAACTAGGATAG
Grin2a	A	AAGACCAGGCCCCAGAGGAGCCATATAGCTTTTCCAATGGTAAAAGAAGGCCTGCCAATGCACTCGATCTTGTCATTTTTTTGCG
	B	CGAAAGCCATGACCTCCGATCACTCCTGGTTGTGCCTTTAGGATTCTGGACGGGCACAGAATTGTTG
Grin2b	A	CACCCTACTGTAATGGCTCTTTCACAAAGCCTTATACGAAGAGCTTTCCCCTGAGGCTGTTAAAGCTGTAGCAACTCTTCCACGA
	B	CGAAAGCCATGACCTCCGATCACTCCCCCTTTCTCACTAACCCTACCGTTAATGACTAAAAACATCCCCCTCTCT
Gria1	A	AACTCGATTAAGGCAACCAGCATGGCCAATCCCAGCCCTCTAGGACGCAAATCACTTGAAGAAGTGAAAGCGAG
	B	CGAAAGCCATGACCTCCGATCACTCTCAAACAGAAACCCTTCATCCGCTTCGACTCGCTACGGGATTTGTAGCAG
Gria2	A	CTTCTTTGACCGTTTTCCTCAGCAAATTAGATCCTCTGCATTTTGAGGCCACGCGATGACGTTCGTCAAGAGTCGCATAATCT
	B	CGAAAGCCATGACCTCCGATCACTCAAAAGTAGAGCATCCACAAAATATCCCCTCGTTTCCCTTTCCTCCTTTTC
Gria3	A	TCAGTGGTGTTCTGGTTGGTGTTGTATAACTGCACAGCAAAGCGGAAAGCCATTTGGAATGATGTGTACTGGGAATAAGACGACG
	B	CGAAAGCCATGACCTCCGATCACTCTATTGGAGGAATCCAAGTGGTCTACGTGGTAGTTCAAATGGAAGGGCTTC
Grm1	A	CTATGTCACTCCACTCGAGGTAACGGATAGTAATGGGCTCACAGCCGCTATGCAGACGAGCTGGCAGAGGAGAGAAATCA
	B	CGAAAGCCATGACCTCCGATCACTCTAGCGTCACGAGGATGCCCAGGCAAGAAAAGGCGATGGCTATGATGGATT

Supplemental Methods

The open field test

The mouse was placed in the corner of a white acrylic open-field box (40 × 40 × 40 cm) and allowed to explore the arena freely for 10 min while its path was monitored and tracked by a video camera, placed 1 m above the center of the arena. The automatic detection of the mouse's traveled distance and the time spent in the central zone (15 cm apart from the walls) was recorded.

The dark-light box test

The dark-light box is an open white rectangle (30 x 20 x 20 cm) attached to a 3 x 3 cm opening to a dark chamber (with a lid and painted in black) (15 x 20 x 20 cm). The light chamber was illuminated at 600 lux. Each mouse was put in the dark chamber, and the latency, as well as the number of visits to the light chamber within 10 min, were measured. Only when all four limbs of the subject crossed the entrance, it was considered as an entry to the light chamber.

The neophobia test

Each subject was placed in an arena with an unfamiliar drink (100 µl sweetened condensed milk) in the center. The mouse was allowed to roam the arena for 10 min and the latency, as well as the number of contacts with the drink, was manually assessed[17].

The burrowing test

The burrowing test is based on the mouse's behavior towards the displacement of items from the tube within its home cage[18]. The tube was filled with 200 g of food pellets covered with 60 g of bedding. The test was performed at 5 p.m. and the pellets remaining in the tube were weighed after 2 h. Then the tube was placed in the cage again. After 12 h, the weight of the remaining pellets in the tube was finally assessed.

The puzzle box test

The puzzle box test was slightly modified from the one described in[20]. The puzzle box consisted of two compartments (a brightly-lit start zone and a smaller covered goal zone) separated by a barrier that had a narrow underpass (about 4 cm wide). Each mouse was introduced into the start zone and the task was to enter the goal zone where it could find some bedding from its home cage. Mice underwent a total of 11 trials over 4 consecutive days, with three trials per day on the first three days, and two trials on the last day. On day 1, during trial 1, the underpass was unblocked and the barrier had an open door above the underpass. In trial 2 and trial 3, the barrier had no doorway and the animals had to enter the goal zone *via* a small underpass. On day 2, trial 4 was identical to trial 2 and trial 3. In trial 5 and trial 6, however, the underpass was filled with sawdust and the animals had to dig through the sawdust. On day 3, the animals had to repeat trial 6 first as well as in trial 7. In trial 8 and trial 9, the animals were presented with the underpass being blocked by a cardboard plug that the mice had to pull out with their teeth and paws to enter the goal zone. Trial 10 on day 4 was again a repetition of trial 9. At the end of the test, in trial 11, the task was to repeat trial 1, like on the first day. After each trial, mice were left for 1 min inside the goal zone.

The fear conditioning test

The fear conditioning test evaluates natural fear learning as described before[21]. For the acquisition session, at first, each mouse had to spend 180 s as habituation in the new arena. Then, an auditory tone was presented for 30 s at a level of 90 dB and frequency of 5,000 Hz with a rise time of 50 ms. A mild foot shock (0.5 mA) was administered during the last 2 s of the tone presentation and co-terminated with the tone. After the shock presentation, an inter-trial interval of 90 s preceded the second and third identical trials. Following the third shock presentation, the mouse remained in the arena for an additional 90 s. On the following day, the contextual testing was conducted similar to the acquisition session including lighting and odor, but without the tone and the foot shock. The experiments lasted for 300 s. On the third day, the cued memory was tested by placing each mouse in a new chamber with different odors, allowing it to habituate for 180 s. The same tone cue as in the acquisition session was then activated for 30 s. Then, an inter-trial interval of 90 s proceeded the second and third trials. The third tone was activated for 300 s until the end of the experiment. The video freeze software was used to record and measure freezing time and numbers.

Amphetamine injection in the open field

Baseline activity in the open field was measured by placing the mouse in the corner of a wooden arena measuring 40 x 40 x 40 cm and allowing it to explore freely for 60 min, while its path was monitored and tracked by a video camera placed 1 m above the center of the arena. Automatic detection of the distance traveled by the mouse as well as its speed was recorded with the SYGNIS tracker software (Sygnis AG). After 1 h, mice were removed from the open field, i.p. injected with 5 mg/kg amphetamine and immediately returned to the open field for another 60 min.

Supplemental references

1. Krestel HE, Mayford M, Seeburg PH, Sprengel R. A GFP-equipped bidirectional expression module well suited for monitoring tetracycline-regulated gene expression in mouse. Nucleic Acids Res. 2001; 29: E39.
2. Jerecic J, Schulze CH, Jonas P, Sprengel R, Seeburg PH, Bischofberger J. Impaired NMDA receptor function in mouse olfactory bulb neurons by tetracycline-sensitive NR1 (N598R) expression. Brain Res Mol Brain Res 2001; 94: 96-104.
3. Hamdan FF, Gauthier J, Araki Y, Lin DT, Yoshizawa Y, Higashi K et al. Excess of de novo deleterious mutations in genes associated with glutamatergic systems in nonsyndromic intellectual disability. Am J Hum Genet. 2011; 88: 306-316.
4. Bahl S, Chiang C, Beauchamp RL, Neale BM, Daly MJ, Gusella JF et al. Lack of association of rare functional variants in TSC1/TSC2 genes with autism spectrum disorder. Mol Autism. 2013; 4: 5.
5. Lin AY, Henry S, Reissner C, Neupert C, Kenny C, Missler M et al. A rare autism-associated MINT2/APBA2 mutation disrupts neurexin trafficking and synaptic function. Sci Rep. 2019; 9: 6024.
6. Wang X, Bey AL, Katz BM, Badea A, Kim N, David LK et al. Altered mGluR5-Homer scaffolds and corticostriatal connectivity in a Shank3 complete knockout model of autism. Nat Commun. 2016; 7: 11459.
7. Castermans D, Volders K, Crepel A, Backx L, De Vos R, Freson K et al. SCAMP5, NBEA and AMISYN: three candidate genes for autism involved in secretion of large dense-core vesicles. Hum Mol Genet. 2010; 19: 1368-1378.
8. Pulido R, Stoker AW, Hendriks WJ. PTPs emerge as PIPs: protein tyrosine phosphatases with lipid-phosphatase activities in human disease. Hum Mol Genet. 2013; 22: R66-76.
9. Nizon M, Cogne B, Vallat JM, Joubert M, Liet JM, Simon L et al. Two novel variants in CNTNAP1 in two siblings presenting with congenital hypotonia and hypomyelinating neuropathy. Eur J Hum Genet. 2017; 25: 150-152.
10. De Rubeis S, He X, Goldberg AP, Poultney CS, Samocha K, Ercument Cicek A et al. Synaptic, transcriptional and chromatin genes disrupted in autism. Nature. 2014; 515: 209-215.
11. Barkus C, Sanderson DJ, Rawlins JN, Walton ME, Harrison PJ, Bannerman DM. What causes aberrant salience in schizophrenia? A role for impaired short-term habituation and the GRIA1 (GluA1) AMPA receptor subunit. Mol Psychiatry 2014; 19: 1060-1070.
12. Zhou QG, Zhu XH, Nemes AD, Zhu DY. Neuronal nitric oxide synthase and affective disorders. IBRO Rep 2018; 5: 116-132.
13. Ding XF, Wang HJ, Qian L, Hu ZY, Feng SF, Wu Y et al. Cpne5 is Involved in Regulating Rodent Anxiety Level. CNS Neurosci Ther. 2017; 23: 266-268.
14. Wang KS, Zuo L, Pan Y, Xie C, Luo X. Genetic variants in the CPNE5 gene are associated with alcohol dependence and obesity in Caucasian populations. J Psychiatr Res. 2015; 71: 1-7.
15. Yang Z, Alvarez BV, Chakarova C, Jiang L, Karan G, Frederick JM et al. Mutant carbonic anhydrase 4 impairs pH regulation and causes retinal photoreceptor degeneration. Hum Mol Genet. 2005;14: 255-265.
16. Goodwin LO, Splinter E, Davis TL, Urban R, He H, Braun RE et al. Large-scale discovery of mouse transgenic integration sites reveals frequent structural variation and insertional mutagenesis. Genome Res. 2019; 29: 494-505.
17. Deacon RM. Hyponeophagia: a measure of anxiety in the mouse. J Vis Exp. 2011: 2613. 10.3791/2613
18. Deacon RM. Burrowing in rodents: a sensitive method for detecting behavioral dysfunction. Nat Protoc. 2006; 1: 118-121.
19. Angebault C, Guichet PO, Talmat-Amar Y, Charif M, Gerber S, Fares-Taie L *et al.* Recessive Mutations in RTN4IP1 Cause Isolated and Syndromic Optic Neuropathies. Am J Hum Genet. 2015; 97: 754-760
20. Ben Abdallah NM, Fuss J, Trusel M, Galsworthy MJ, Bobsin K, Colacicco G et al. The puzzle box as a simple and efficient behavioral test for exploring impairments of general cognition and executive functions in mouse models of schizophrenia. Exp Neurol. 2011; 227: 42-52.
21. Humeau Y, Reisel D, Johnson AW, Borchardt T, Jensen V, Gebhardt C et al. A Pathway-Specific Function for Different AMPA Receptor Subunits in Amygdala Long-Term Potentiation and Fear Conditioning. The Journal of Neuroscience. 2007; 27: 10947.
22. Kang J, Park H, Kim E. IRSp53/BAIAP2 in dendritic spine development, NMDA receptor regulation, and psychiatric disorders. Neuropharmacology 2016; 100: 27-39.
